# Fibronectin in Cancer: Friend or Foe

**DOI:** 10.3390/cells9010027

**Published:** 2019-12-20

**Authors:** Tsung-Cheng Lin, Cheng-Han Yang, Li-Hsin Cheng, Wen-Tsan Chang, Yuh-Rong Lin, Hung-Chi Cheng

**Affiliations:** 1 The Institute of Basic Medical Sciences, College of Medicine, National Cheng Kung University, Tainan 70101, Taiwan; s58044012@mail.ncku.edu.tw (T.-C.L.); s58021014@mail.ncku.edu.tw (C.-H.Y.); s58031043@mail.ncku.edu.tw (L.-H.C.); wtchang@mail.ncku.edu.tw (W.-T.C.); 2 Department of Biochemistry and Molecular Biology, College of Medicine, National Cheng Kung University, Tainan 70101, Taiwan; s16064107@mail.ncku.edu.tw

**Keywords:** fibronectin, pericellular fibronectin assembly, tumor suppression, cancer metastasis, extracellular matrix, senescence, hypoxia, epithelial–mesenchymal plasticity, circulating tumor cells, premetastatic niche

## Abstract

The role of fibronectin (FN) in tumorigenesis and malignant progression has been highly controversial. Cancerous FN plays a tumor-suppressive role, whereas it is pro-metastatic and associated with poor prognosis. Interestingly, FN matrix deposited in the tumor microenvironments (TMEs) promotes tumor progression but is paradoxically related to a better prognosis. Here, we justify how FN impacts tumor transformation and subsequently metastatic progression. Next, we try to reconcile and rationalize the seemingly conflicting roles of FN in cancer and TMEs. Finally, we propose future perspectives for potential FN-based therapeutic strategies.

## 1. Introduction

**Figure 1:**
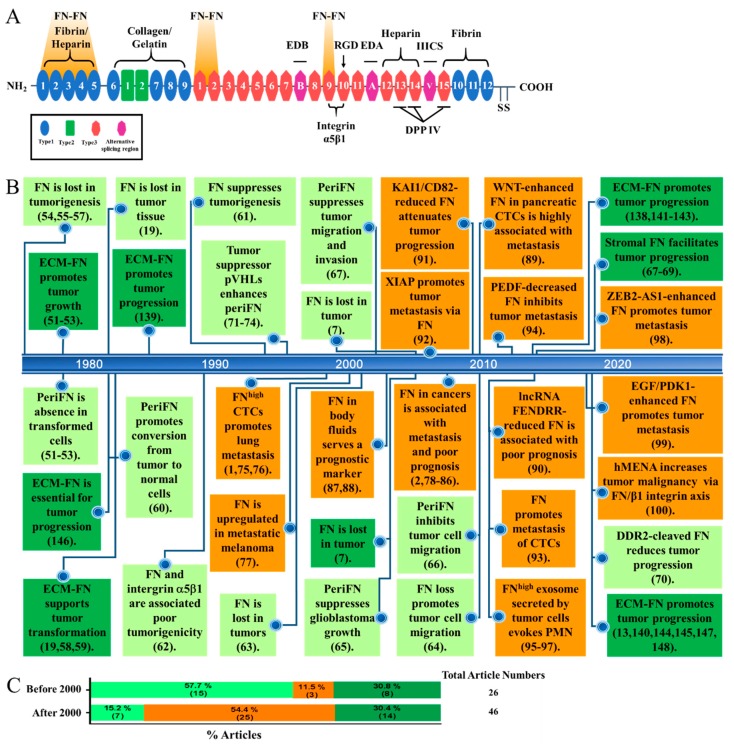
(**A**) The structure of fibronectin (FN) containing three types of repeats and three alternative splicing regions (EDA, EDB, and IIICS) with several well-known binding sites for extracellular matrix (ECM) components (fibrin, heparin, collagen, and gelatin), polymeric assembly (FN–FN), cell adhesion (integrin α5β1), DPP IV, and two C-terminal disulfide bonds for dimeric FN. (**B**) Publications in recent some forty years regarding the roles of cancerous FN and stromal FN in ECM in tumor progression as represented in a time-line pattern. Among 26 publications before 2000, 15 (57.7%) papers are related to the role of cancerous FN in tumor suppression (in light green boxes), 3 (11.5%) papers are related to the role of cancerous FN in metastasis promotion (in orange boxes), and 8 (30.8%) papers are related to the role of stromal FN in promoting early tumor progression but not late metastasis (in dark green boxes). On the contrary, Among 46 publications after 2000, 7 (15.2%) papers are related to the role of cancerous FN in tumor suppression (in light green boxes), 25 (54.4%) papers are related to the role of cancerous FN in metastasis promotion (in orange boxes), and 14 (30.4%) papers are related to the role of stromal FN in promoting early tumor progression but not late metastasis (in dark green boxes). Abbreviations in boxes are referred to the context of this article. (**C**) Percentages of articles for the three various roles of FN (the same colors as depicted in (**B**) before 2000 and after 2000. Numbers in the parenthesis represent article numbers.

Identification of a general and suitable target that is unambiguously oncogenic or tumor suppressive is a foundation on which cancer therapeutic strategies could be designed and developed. Fibronectin (FN) (Figure 1A) has long been proposed to play an important role in the pathobiology of cancer. Numerous studies have indeed provided possibilities to target FN for fighting against cancer [1,2,3,4,5,6]. However, the role of FN in tumorigenesis and malignant progression has been highly controversial [7,8]. On the one hand, it has been reported that FN expression in tumor cells plays a tumor suppressive role to prevent tumor transformation and to halt their early progression [9]. On the other hand, abundant evidence reveals that FN provokes late stages of cancer metastasis and is associated with poor prognosis when endogenously expressed in tumor cells. When deposited into extracellular matrices (ECMs) in the immunosuppressive tumor microenvironments (TMEs) in which tumor cells are often the driving force to induce inflammatory responses, FN promotes early tumor progression [10,11,12,13,14,15] but is paradoxically correlated with a better prognosis [7,16,17,18,19] (Figure 1B,C). Before resolving such obviously paradoxical roles of FN in cancer development, it is of high risk to simply target FN for controlling cancer. In this review article, we will first delineate how FN paradoxically impacts the pathobiology of cancer. Next, we will try to reconcile and rationalize the seemingly conflicting roles of FN in cancer. Finally, we will provide future perspectives by proposing potentially suitable FN-targeting therapeutic strategies.

## 2. The Pathobiology of Cancer

### 2.1. Transformation

**Figure 2:**
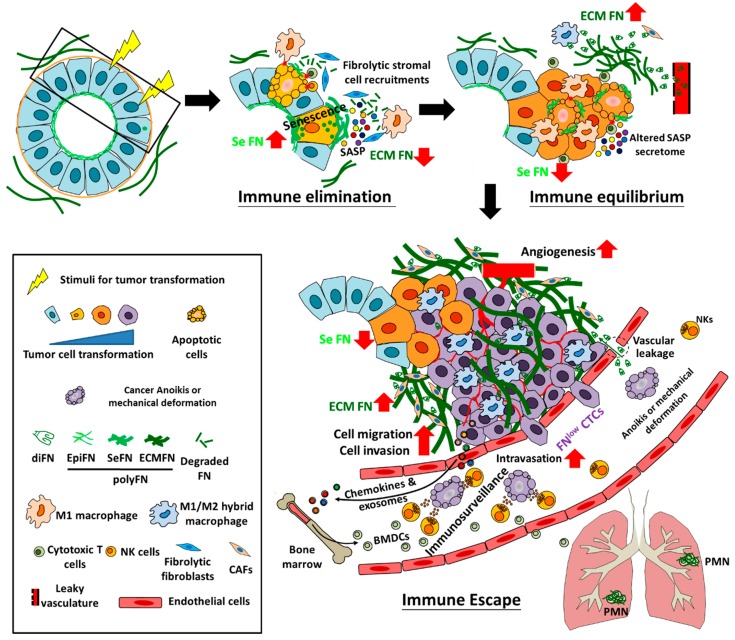
Hypothetic illustration of tumor transformation and early progression involving immunoediting in which FN participates. During tumor transformation and early progression, moderate FN-expressing normal cells, most often epithelial cells, first enter the senescence state under oncogenic stimuli (including oncogenic activation, loss of tumor suppressor genes, and diverse factors derived from environments), in which endogenous FN expression is highly promoted and senescence-associated secretory phenotypes (SASPs) are subsequently secreted by those senescent cells to recruit fribrolytic pro-M1 macrophages and fibroblasts, cytotoxic T cells, and natural killer cells (NKs) for degrading and remodeling ECM (including ECM FN) and clearing damaged or senescent cells (at the immune elimination stage of 3E). Progressing from the immune equilibrium to the immune escape stage, tumor cells are transformed from the survived stimulus-induced senescent cells and slowly evolve to conquer immunosurveillance in the primary tissues, during which endogenous FN is drastically downregulated, whereas tumor cell-driven FN deposition in the ECM of TMEs is greatly elevated, resulting from plasma FN extravasated from leaky vasculature and secretion (most likely the altered SASP secretome) by M1/M2 hybrid macrophages and cancer associated fibroblasts (CAFs). Eventually, tumor cells increase their migratory and invasive activities, trigger angiogenesis, outgrow in the primary tissues, and intravasate into the nearby blood vessels to become FN^low^ CTCs, which, although in large amounts, suffer greatly from anoikis, mechanical deformation, and immunosurveillace (majorly via NK cells) in the circulation. Nevertheless, the fast growing tumor cells in the primary tissues can secrete chemokines and exosomes to drive bone marrow-derived cells (BMDCs) that are recruited in the distant organs (e.g., the lungs) to establish premetastatic niche (PMN) where macrophages (most likely M1/M2 hybrid as in the primary tissues) and CAFs are mainly responsible for the FN deposition in the ECM of PMN to prepare a suitable TMEs for the future extravasated metastatic DTCs. diFN: dimeric form of FN; EpiFN: FN expression in normal epithelial cells; SeFN: FN expression and surface assembly in the stimuli-induced senescence cells; ECMFN: FN deposition in ECM.

Accomplishment of cancer development, a rather slow and chronic process, temporally and spatially requires various cellular activities across different tissues. Tumor cells originate from healthy, often epithelial, cells that acquire hereditary mutations [20] or somatic mutations in response to a diversity of environmental stresses. Owing to self-defense, healthy cells harboring first fit of oncogenic activation or tumor suppressor gene (TSG) inactivation become senescence instead of continued oncogenic proliferation until a second hit of somatic mutation occurs, illustrated as the Knudson’s “two-hit” model [21,22]. As such, once these senescent precancerous cells are transformed, they possess intratumor heterogeneity due to genomic instability caused by the abnormally released cell cycle progression [23,24] (Figure 2).

### 2.2. Early Progression

In contrast to such intratumor heterogeneity, immune and stromal cells in the tumor microenvironments (TMEs) initially arise to eliminate pre-tumor abnormal cells and early transformed cells that encounter environmental stresses. Consequently, these stresses impose early transformed cells enormous selective pressures to force evolution, rendering tumor cells staying in the equilibrium stage for a long while before being able to escape immunosurveillance and make a malignant progression [25,26,27]. However, these events are seemingly insufficient to warrant cancer metastasis, an event highly associated with hypoxia within TMEs, a lethal environment when tumor size continue to increase without sufficient oxygen and nutrient supplies [28] (Figure 2).

### 2.3. Hypoxia at the Crossroad between Early Progression and Late Malignancy

**Figure 3:**
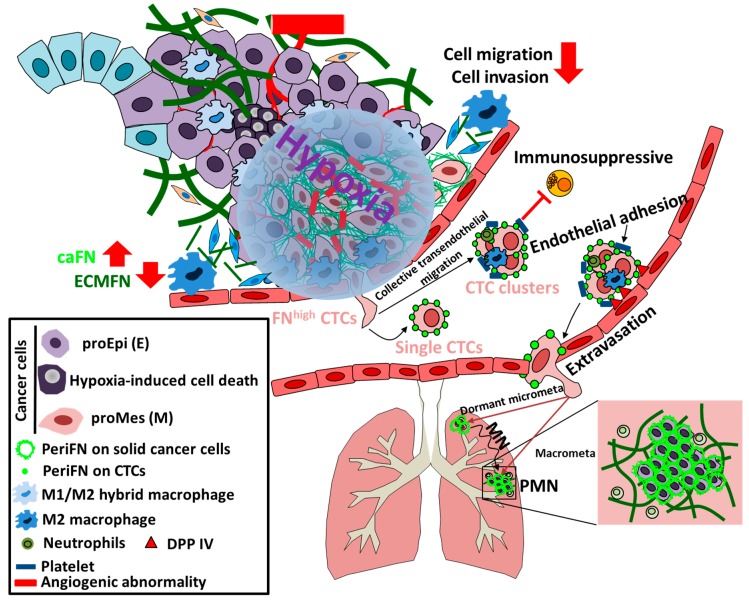
Hypoxia is at the cross road likely to decide whether tumor cells could reexpress FN and pave the way for themselves to distant organs. Once the outgrown tumor cells rich a certain size, oxygen concentration within the TMEs becomes significantly less which forms an enormous harsh environmental pressures to force cell death of the majority of outgrown tumor cells due to hypoxia with only a small number of cells continue to evolve and survive (most likely due to slow cycling and cancer stemness). These slow cycling stem-like cells drastically reexpress endogenous FN in a HIF1α-dependent or -independent manner. At the same time, the pro-epithelial (proEpi (E)) phenotypes of tumor cells are reversed into pro-mesenchymal ((proMes (M)) phenotypes and M1/M2 hybrid macrophages and cancer associated fibroblasts (CAFs) are activated to become fibrolytic CAFs, leading to significant clearance of ECM FN. While angiogenesis is promoted by hypoxia with abnormality, the abilities of the FN^high^ tumor cells to migrate, invade, and intravasate are greatly reduced. Although the numbers of intravasated FN^high^ circulating tumor cell (CTC) are reduced, CTC clusters can be present via collective transendothleial migration in addition to single CTCs to enhance their resistance to circulatory stresses, e.g., anoikis, mechanical deformation, and immunosurveillance (majorly via NK cells). CTC clusters, that are often formed together with platelets, M2 macrophages, or neutrophils, can adhere to endothelia via binding to dipeptidyl peptidase IV (DPP IV) by periFN assembled on FN^high^ CTCs, followed by extravasation and migration to either non-PMN locations or PMN which has early been established by the outgrowing tumor cells in the primary tumor cells. The PMN-lodged proMes DTCs tend to be reversed to proEpi again and quickly form macrometastasis (Macrometa), whereas the non-PMN-lodged proMes DTCs first enter a dormancy and only exhibit micrometastasis (micrometa) for a long period of time until metastatic nich (MN) is established by the proMes DTCs, which in turn reverse DTCs to proEpi without compromising FN expressing and lead to the outgrowth of DTCs into macrometastasis.

Oxygen delivery within the TMEs is inefficient mainly due to various abnormalities in the tumor vasculature [29]. Indeed, the stable expression of the hypoxia-inducible transcription factors HIF-1 and HIF-2 under hypoxia are highly correlated with increased distant metastasis and poor prognosis [28]. Hypoxia has been reported to contribute to premetastatic niche (PMN) [30,31], immune evasion [32], and survival in distant tissues [28], events required for the accomplishment of cancer metastasis in distant organs [28] (Figure 3).

### 2.4. Tumor Cell Survival in the Circulation

The liberation and survival of circulating tumor cells (CTCs) is a requirement for the colonization of disseminated tumor cells (DTCs) in secondary organs and finally outgrowth and macrometastasis development [33,34]. Like those in the primary tissues, CTCs are also heterogeneous and only certain subpopulation is able to make their way to final metastatic growth [35]. Accumulating evidence indicates that hypoxia-induced EMT allows the intravasated CTCs to survive, most likely via resistance to detachment-induced anoikis and/or NK-mediated immunosurveillance, and to colonize distant organs [36,37] (Figure 3).

### 2.5. Premetastatic Niche and Macrometastasis Outgrowth

Serious attention has recently been paid to the concept of PMN which plays an important role in the outgrowth of dormant DTCs into macrometastatic tumor lesions in which EMT could be reversed into mesenchymal–epithelial transition (MET) [38,39]. PMN is established in the distant organs mainly through effects of VEGFR^+^ bone marrow-derived cells (BMDCs) that have been driven by cytokines or exosomes secreted by the metastatic competent tumor cells in primary tissues long before extravasation of DTCs in distant tissues [40,41]. Obviously, the reason of DTCs to be reversed from EMT to MET within PMN is that DTCs in MET state adapt better the growth conditions in distant tissues which are otherwise disfavoring the outgrowth of DTCs in EMT state [38,42,43,44] (Figure 2 and Figure 3).

## 3. The Paradox of the Role of FN in Cancer

### 3.1. Structure and Functions of FN

FN was originally identified a cell surface glycoprotein that is present on the cell surfaces of nontransformed cell lines [45]. FN mRNAs, about 8-kb, encode for FN protein subunits, dependent of alternative splicing, with a range in size from 230–270 kDa [46]. FN harbors three repeating units, name as types I, II, and III repeats to contain various binding sites for collagen/gelatin, integrins, heparin, FN, and other extracellular molecules. Numerous folded structures in each type I and II FN repeat are stabilized by two intramolecular disulfide bonds, whereas type III FN repeats are seven-stranded β-barrel structures without any disulfide bond. The minimal ~500-kDa dimer form of FN is further stabilized by two intermolecular antiparallel disulfide bonds at the C terminus of each monomer (Figure 1A) [47,48]. Polymerization and pericellular FN matrix assembly (periFN) engaging disulfide bond-dependent and -independent dimerization of FN subunits, self-binding activity that associates FN dimers into fibrils, and cell-binding activity enable FN to participate various physiological and pathological functionalities. Illustrations regarding detailed biology of FN can be referred to other review articles [49,50].

### 3.2. FN Plays a Tumor Suppressive Role in Tumor Cells but Serves as an Oncogenic Factor in the Surrounding Stromal Tissues

Before the early 1990s, while stromal FN in TMEs favors the growth of tumor cells, cancerous FN has unambiguously been deemed as a tumor suppressive factor [51,52,53]. In line with these findings, it has been found that, by comparing lots of non-transformed and tumorigenic epithelial cell lines inoculated in nude mice [54], loss of FN expression and cell surface matrix assembly is correlated with malignant transformation [54,55,56]. Clinically, immunohistochemistry (IHC) staining reveals that no FN can be detected in most of the carcinoma tumor cells, whereas the surrounding stromal tissues are strongly positive, echoing the above-described pre-clinical findings [19]. Other evidence also shows that the level of periFN is reduced or it is absent on transformed cells [57,58,59]. Restoration of periFN on rat kidney transformed cells convert them into extremely flat cells with abundant fibrillar FN on cell surfaces and these cells subsequently grow into monolayer with normal morphology, implying that periFN expression indeed plays a tumor suppressive role [60]. Overexpressing a recombinant FN clearly suppresses transformed phenotypes of human fibrosarcoma cells [61]. Consistently, it has been found that overexpressing FN receptor integrin α5β1 in Chinese hamster ovary (CHO) cells induces more periFN deposition, rendering CHO cells significantly less migratory and anchorage-independent [62]. Despite the fact that drastically fewer reports document FN as a tumor suppressor after 2000s, there remain several findings characteristic of tumor suppression for cancerous FN [7,63,64,65,66,67]. The impact of high stromal FN expression to which tumor cells directly adhere in early malignant tumor progression may be due to the induction of matrix metalloproteinases (MMPs) in tumor cells, which facilitates tumor migration, invasion, angiogenesis, and intravasation [68,69]. During the TWIST1-promoted ovarian cancer metastasis, discoindin domain receptor 2 was upregulated to increase activity of matrix modeling enzymes and the cleavage of FN, leading to elevated migratory and invasive activities of tumor cells [70]. Mechanistically, periFN serving as a tumor suppressor has further been evidenced by the fact that the ability of tumor cells to assemble periFN is abolished due to the mutation and deficiency of an intimate FN-binding partner von Hippel-Lindau proteins (pVHLs) [71]. However, periFN remains deficient in many other types of tumors with intact pVHLs, suggesting that loss of FN expression or periFN deficient may also be regulated by VHL-independent pathways. Alternatively, aberrant functions of yet-to-be-identified factors, e.g., NEDD8 that modifies pVHL or elongin C, elongin B, cullin 2, or RBX1 in the pVHL complex [72,73,74] may be responsible for the tumor cells losing the ability to assemble periFN (Figure 1B).

### 3.3. FN Promotes Cancer Metastasis

Accumulating evidence has emerged to reveal that cancerous FN expression critically contributes to tumor malignancy, metastasis, and patients’ poor prognosis since early publications controversially claim that periFN serves as an endothelia-binding ligand on blood-borne tumor cells to mediate and promote lung colonization and metastasis [1,75,76,77]. Numerous reports have also employed either genetic or proteomic approaches to demonstrate that cancerous FN expressions in various types of tumor cells are indeed experimentally and clinically associated with tumor malignancy, metastasis, or poor prognosis [2,78,79,80,81,82,83,84,85,86]. Higher levels of plasma, serum, or urine FN expression could be detected in late stages and metastatic renal cell carcinoma or colorectal cancer patients and may clinically serve as excellent non-invasive prognostic biomarkers [87,88]. In line with such metastatic-promoting role of cancerous FN, FN is highly expressed in the pancreatic CTCs that possess a high metastatic potential empowered by the WNT signaling [89]. In many findings elaborating molecular mechanisms underlying distant cancer metastases, cancerous FN expression has often identified as a critical mediator. For example, FN expression mediates the metastatic promoting role of the decreased expression long non-coding RNA FENDRR, which is associated with poor prognosis of gastric cancer [90]. In addition, the mechanism underlying the anti-adhesion effect of metastatic suppressor KAI1/CD82 is that overexpressing KAI1/CD82 results in a reduced FN expression, leading to an attenuation of the matrix adhesion of human prostate cancer cells [91]. In the cancer metastasis that is mediated by the interaction between X-linked inhibitor of apoptosis (XIAP) and survivin but independent of inhibition of cell death, FN expression serves a downstream signaling factor to promote the metastasis process [92]. Furthermore, FN expression has often been found to mediate various metastatic-promoting mechanisms [93,94,95,96,97,98,99,100] (Figure 1B).

## 4. Reconciliation of the Paradoxical role of FN in Cancer

**Figure 4:**
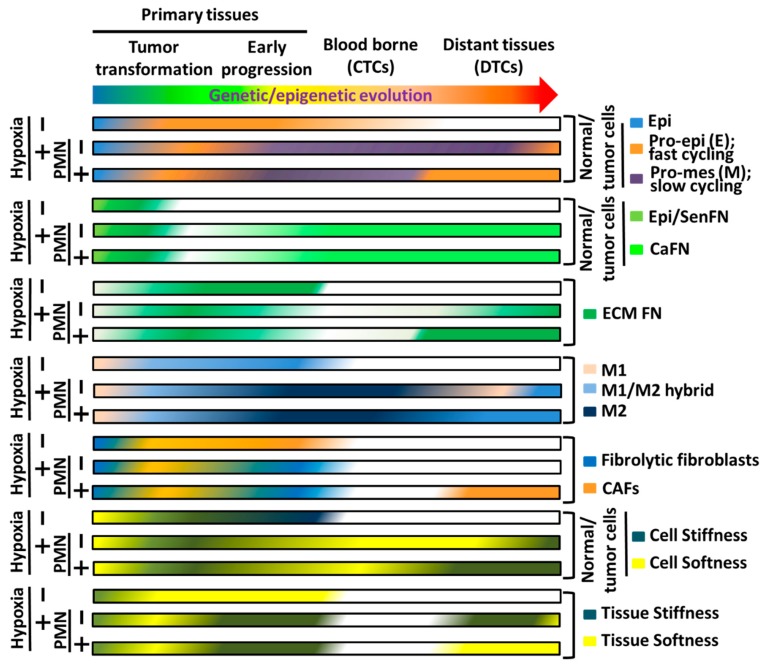
Hypothetic illustration of the interchanges of various phenotypes in tumor cells, stromal cells, caFN, and ECM-FN as exhibited in Figure 2 and Figure 3 during the whole processes of tumor genetic/epigenetic evolution and progression where hypoxia and PMN are critically at the crossroads. “−” means in the absence and “+” means in the presence.

Attention has particularly been paid to the fact that, with a few exceptions [101,102,103,104,105,106], the role of FN expression in tumor cells was primarily thought of as a tumor suppressor prior to the 1990s, yet later the consensus switched to its role being a metastatic promoter as aforementioned. It remains obscure why there are such two cut-off phases as to the different time periods for the obviously conflicting mainstream conceptions regarding the role of FN in tumor progression. Most fascinatingly, not only have almost no rebuttals been made from either side, but when one side deems FN to be a tumor suppressor or metastatic promoter, the effect of FN in tumor metastasis or tumorigenesis, respectively, has often not been tested (Figure 1B). This implies the possibility that all the findings are truthful but difficult to rationally reconcile given that the roles FN plays during tumor progression are too complicatedly intertwined. Here, several possibilities are proposed for the seemingly paradoxical roles of FN in cancer progression (Figure 2, Figure 3 and Figure 4).

### 4.1. The Role of FN Expression in Epithelial Cell Senescence and Tumor Transformation

During early process of transformation, normal cells usually encounter various types of stresses, leading to premature cellular senescence that autonomously halts the continuous growth of damaged cells to prevent the occurrence of tumor and non-autonomously affects the surrounding microenvironments, including immune cell recruitment for eliminating the senescent cells (so called elimination stage of immunoediting during tumor progression) and stromal cell senescence for tissue remodeling [26,107,108,109,110]. Cumulative evidence indicates that increased FN expression is a relatively common event in the senescent cells and even in the secretome of ASAP [111,112,113,114]. Indeed, FN upregulation has been deemed as one of prominent senescent marker [113,115] (Figure 2). It is reasonable to associate FN overexpression with premature cellular senescence that is triggered by a variety of stresses, including ER stresses, due to tumor transformation factors, e.g., oncogene-induced senescence (OIS) or senescence induced by loss of TSG, as FN is a large and highly structured ECM glycoprotein synthesized in the ER with a molecular weight of a disulfide-bonded dimer as high as ~500 kDa [50] and may easily suffer structural misfolding due to diverse reasons including mutations, inadequate stoichiometric amounts of oligomerization partners, shortage of chaperone availability, increase in nascent client proteins, nutrient deprivation, viral infection, hypoxia, and oxidative stress [116]. The newly synthesized FN monomer entering the lumen of the ER undergoes proper folding, posttranslational modifications, followed by the acquisition of disulfide bonds before it is in the appropriate structure to execute physiological functions [117]. Normally, when FN is misfolded during synthesis in the ER, it may be exported from ER back to cytosol for the degradation by the ubiquitin/proteasome system, a process named as the ER-associated protein degradation (ERAD) [116] and the cell integrity ensues. However, when FN is overexpressed with too many misfolded FN molecules exceeding the affordable ERAD, ER stress is likely to be spawned, resulting in cell death or premature cellular senescence [109,118].

The possibility that FN overexpression accounts for normal epithelial senescence caused by various types of stresses [118,119,120,121,122,123] is substantially supported by the findings that silencing FN transcription or depleting periFN of pre-cancerous cells or pre-malignant tumor cells promotes characteristics of tumor progression, including proliferation, migration/invasion, tumor sizes, anchorage-independent cell growth, and angiogenesis [53,64,65,66,67,106,124,125,126,127]. Whether the promotion of tumor malignancy by FN depletion is due to the suppression of senescent phenotypes warrants further investigation. PeriFN suppresses tumor progression could further be evidenced by the fact that, in von Hippel-Lindau (VHL) disease, defected pVHL protein gives rise to renal tumorigenesis where periFN assembly is hampered. Cells that are deficient in VHL gene are less competent to eliminate misprocessed proteins [128], possibly disrupting the binding of CUL2 to the VHL-elongin complex [72,129]. CUL2 is believed to target misprocessed proteins for ubiquitination and subsequent clearance by protein degradation [130]. Conceptually, cells with pVHL-deficient may not properly assemble periFN on their surfaces but accumulate misfolded FN in the ER lumen, leading to unmanageable ER stress and premature cellular senescence [131]. Such senescent cells may be maintained in an equilibrium stage until they are able to significantly downregulate FN, reduce ER stress, evade the cellular senescence, and progress to the escape stage (Figure 2) [26]. In line with the aforementioned idea of the molecular mechanism underlying cellular senescence escaping and tumor transformation, HSP90 chaperon proteins have been reported to be capable of binding to and guiding newly synthesized FN in the ER lumen of normal epithelial cells, facilitating proper folding of glycosylated FN structure followed by appropriate periFN assembly on cell surfaces [132,133]. When normal epithelial cells are treated with HSP90 inhibitors, cells also suffer senescence due to a reduced ability to properly guide the folding of FN for a correct periFN assembly, unavoidably leading to cellular senescence and more FN is expressed as a senescent marker [132]. Indeed, HSP90s are deemed as an essential chaperon family to prevent senescence of normal epithelial cells [134]. The HSP90 deficiency-induced senescence may be evaded if endogenous FN synthesis is prohibited, rationalizing why, in order to allow transformed tumor cells to continue proliferating, they must downregulate their endogenous FN synthesis to foster the regrowth of senescent cells to become transformed tumor cells when the genome instability reaches the degree to which cellular senescence can be evaded and the regrown senescent cells become tumorigenic [20,107,135,136].

### 4.2. ECM-Deposited FN Derived from Plasma, CAFs, and Macrophages during Inflammation Promotes Tumor cell Growth, Migration, Invasion, Angiogenesis, and Intravasation

The mutual interactions between parenchymal cells that endure the process of oncogenic stresses and transformation and stromal microenvironments harboring mesenchymal cells endow tumorigenesis and tumor progression [26,137]. In contrast to the significant downregulation of FN in tumor cells in primary tumor tissues, FN that is deposited in the ECM of TMEs plays an important role in promoting tumor progression, including tumor cell growth, migration, invasion, angiogenesis, and intravasation [13,138,139,140,141,142,143,144,145,146,147,148]. However, how FN production and FN matrix deposition and organization are regulated by tumor cells has been less understood. Here, we attempt to propose possibilities. The ECM-depositing FN can be derived from either plasma [149] or various cell types, of which fibroblasts and macrophages have especially drawn attention [136,145,150] (Figure 2). Endothelial cells and their cell-to-cell junctions are essential in maintaining vascular integrity and vascular permeability to plasma and cells [151]. In certain physiological circumstances, endothelial cell junctions may be transiently dismantled, allowing inflammatory leukocytes and plasma proteins including FN to enter a wound tissue for a protective barrier against invading microorganism, prevention of severe water loss, clearance of damaged cells, and healing of the wound [152,153]. However, when endothelia are situated within tumor tissues where tumor cells release factors to trigger angiogenesis with altered junctional compositions [154,155], hyperpermeability of tumor vessels renders pro-tumor leukocyte recruitment and persistent influx of plasma molecules, e.g., FN and fibrin, into chronic inflammatory TMEs, consequently facilitating tumor growth, migration/invasion, and intravasation [149,153,156,157,158]. Fibroblasts surrounding tumor cells are generally considered as the main professional matrix producers called CAFs [145,159,160]. In the beginning of oncogenic processes, pro-inflammatory cytokines in non-autonomous SASP [113,161], produced and secreted by the senescent parenchymal cells that suffer diversity of stresses, are essentially responsible for the alteration of surrounding microenvironments, including fibroblast activities [162,163]. While fibroblasts are thought to be temporarily senescent to control fibrogenesis in the neighbor stroma and recruit pro-inflammatory cells to clear the damaged senescent cells, the transient fibrolytic senescent fibroblasts are altered to be persistent FN-generating fibrogenic fibroblasts in response to the altered secretome of tumor cells that evolve to escape immunosurveillance during tumor transformation [107,145,164]. The fact that persistent senescent fibroblasts surrounding transformed tumor cells aggravate FN-enriched fibrosis [145] is substantiated by the findings that the fibroblast senescence induces myofibroblast differentiation in a paracrine manner to express a profibrotic SASP and eminent FN expression and that reduction of fibrillar FN formation ameliorates organ fibrosis [144,153,165]. It has been known that FN and enzyme LOX critically contribute to fibrosis in various tissues [166,167]. Consistently, SASP of fibrogenic senescent fibroblasts, although contains decreased ECM components, e.g., collagens and elastin [168,169,170], exhibits elevated FN and LOX [127,171], implying that long-term senescent fibroblast-synthesized FN critically contributes to the ECM deposition within TMEs. Another pro-inflammatory cell type that is able to contribute to FN deposition in TMEs is macrophage [172,173,174,175,176]. It has been reported that alternatively activated macrophages do express FN [177], facilitating tumor progression, migration, invasion, and intravasation [172,178,179,180]. Additionally, macrophages have been reported to be capable of remodeling FN-containing ECM matrix deposition and driving pro-malignant interactions between tumor cells and ECM in the TMEs [181,182] Clinically, more than 80% of all meta-analytic results demonstrate a strong association between the presence of macrophage and patients’ poor prognosis in various types of cancer [183,184]. Indeed, the poor prognosis for cancer patients has been attributed to high expression levels of CSF-1, the major lineage regulator for macrophages [185,186]. Importantly, CSF-1 has been evidenced as a regulator of endogenous cellular FN expression [187,188]. Furthermore, during the hepatocellular carcinoma development, oncogene-induced senescent hepatocytes secrete SASP cytokines, e.g., CCL2, to exert pro-tumor effects [136]. CCL2 secreted by tumor cells is well known to be a crucial chemokine factor for immature monocyte recruitment [186] and associated with poor prognosis [189], whereas its absence is associated with increased survival in cervical cancer patients [190]. Additionally, CCL2, when engaged with CCR2 expressed on the recruited immature monocytes and triggering their maturation into tumor associated macrophages (TAM), has been reported to evoke IL6 secretion that activates STAT3-mediated signaling [191]. FN expression and secretion has well been known to be provoked by IL6-STAT3 signaling axis [192,193]. These findings suggest that TAMs may significantly contribute to persistent FN secretion and deposition within TMEs, facilitating tumor progression.

Whereas it is beyond the shadow of doubt that macrophages in the tumor-induced inflammatory milieus can deposit and organize the FN matrices into TMEs [172,176,177], it remains obscure whether a specific macrophage population contributes to such FN matrix deposition. It is probably due to a fact that myeloid monocyte-derived macrophages are relatively pliable and not only limited to the M1/M2 conversion in response to diverse stimulatory cues [194,195,196]. For instance, FN matrix deposition, instead of provisional FN, has been deemed as one of markers in alternatively activated macrophages often designated as M2 macrophages within the pro-tumor inflammatory TMEs [197], whereas it has conversely been revealed that macrophages characteristic of the M1 type responsible for clearing damaged cells during the elimination stage of tumor transformation remain M1 type and are not converted to M2 type [198]. Indeed, classification of macrophages has also evolved originally from dichotomy to macrophage spectra before macrophage circle [196]. The bottom line for the role of macrophages and their secretion of FN eventually organized into FN matrices in pro-tumor TMEs is that any type of macrophages belonging to TAMs should comply with the criteria in which macrophages are potent to persistently secrete FN and render FN matrix deposition in TMEs in favoring tumor growth and malignant development when endogenous FN expression is downregulated in tumor cells. However, how these two mutually cross-interactions between tumor cells and macrophages are engaged deserves further investigations.

### 4.3. Hypoxia-Induced Reexpression of FN in Tumor Cells and Cancer Metastasis

As low FN expressing (FN^low^) tumor cells that bypass senescence effects at the elimination stage and drive persistent FN deposition in the ECM within TMEs to facilitate tumor growth and progression prosperously proliferate and enlarge tumor sizes up to ~100–200 μm away from blood vessels, the low oxygen pressure within the TMEs renders hypoxia due to insufficient blood vessel supply [199,200,201,202]. The hypoxic microenvironments give an enormous impulse to tumor cells as well as stromal cells metabolic threatening, eventually leading to apoptosis of tumor cells that are sensitive to prolonged hypoxia [203,204]. Due to the entities of genomic instability and heterogeneity in tumor cells, some tumor cells, however, adapt to hypoxia, survive, and become highly metastatic [199,202] (Figure 3). Hypoxia-induced factors (HIFs), e.g., HIF1 and HIF2, are among molecules upregulated in the tumor cells surviving the hypoxic environments the most studied genes as master regulators [28,205]. Interestingly, endogenous FN synthesis is one of consequences of HIF activation [206,207,208,209]. Importantly, HIFs are highly instable under normoxia mainly due to the oxygen-dependent hydroxylation of pVHL-binding proline residues in HIFs by proline hydroxylase (PHD), triggering specific binding of pVHL, an E3 ubiquitin ligase capable of rendering polyubiquitination and proteasomal degradation of HIFs [28,210]. In cancer patients with VHL mutations, HIFs are always stabilized [211]. It has been reported that pVHL is required for periFN assembly [71,131,212], perfectly in line with the concept that FN expression and periFN assembly serve as tumor suppressing factors [54,56]. Consistently, in the early tumor progression, FN has always been found to be downregulated in prosperous tumor cells in primary tumor tissues [19,54,55,56]. These results suggest that periFN assembly is disabled in VHL-deficient tumor cells in the presence of stable HIFs. Since it has well been documented that FN expression in tumor cells is highly correlated with metastatic potency and poor prognosis, upregulation of endogenous FN in metastatic cancer cells of VHL-deficient patients may conceptually due to HIF-independent molecular mechanisms, warranting further investigation. This hypothesis is supported by the findings that FN can still be significantly upregulated in in the mouse embryonic fibroblasts (MEFs) isolated from HIF-1α–/– mice under hypoxic conditions [213].

Abundant evidence indicates that epithelial–mesenchymal (E–M) plasticity is a cellular activity essential to cancer cells that are competent to metastasize in distant organs [36,214,215]. Moreover, E–M plasticity has long been shown to regulate tumor- or metastasis-initiating characteristics of tumor cells [216,217,218]. Interestingly, cancer stemness has also been documented to contribute to resistance to anti-cancer drugs [219,220]. FN is one of most studied genes that are upregulated in tumor cells bearing E–M plasticity and often employed as a biomarker for the mesenchymal phenotype [221,222]. Hypoxia is often deemed as a stimulator for tumor cells to be switched from epithelial to mesenchymal phenotype and become cancerous stem cells [216,223]. Altogether, these findings fully support that FN can be reexpressed in tumor cells under hypoxic conditions either by HIF1α-dependent or -independent signaling regulations. The metastatic-initiating and drug-resistant characteristics of these FN-reexpressing hypoxia-altered tumor cells may endow them abilities to progress toward secondary growth in distant organs. It is worth researching as to whether FN reexpression in hypoxia-altered tumor cells is a cause or a consequence of metastatic initiation and drug resistance. Like stress-induced cellular senescence of precancerous cells, hypoxic stresses may also impulse apoptotic and senescent pressure to the prosperously growing tumor cells [224,225]. Indeed, the majority of tumor cells die due to apoptosis under hypoxic conditions [226]. Within heterogeneous populations, the outgrowing tumor cells in primary tissues may suffer again from senescent stimulation under hypoxic conditions [225]. In line with such thought, senescence biomarkers such as FN reexpression and RhoA-mediated actin stress fiber cytoskeleton become apparent in tumor cells suffering from hypoxic stimulations [206,227,228]. Conceptually, tumor cells that have continuously evolved and harbored certain degree of genomic instability would not behave similarly to precancerous cells and stay in the senescent state where cells are stopped in the G0 phase but likely slower their cell cycle progression to avoid apoptosis and are prepared to switch to mesenchymal plasticity [202,216,217]. Indeed, the FN-reexpressing tumor cells under hypoxic conditions are potent to become metastatic-initiating cells and more resistant to anti-cancer drugs [216,218]. Consistently, numerous publications have unveiled that once tumor tissues ensuing hypoxic environments upon therapeutic treatments, tumor cells that acquire drug resistance develop mesenchymal plasticity and metastasis-initiating capability [229,230,231,232]. Most importantly, it has been demonstrated that, likely due to XIAP-dependent pathway, drug-resistant tumor cells often highly express endogenous FN and are highly metastatic under hypoxic conditions [92,233,234]. Accumulating evidence indicates that cancer stemness and drug resistance do make tumor cells grow significantly slower [235,236]. These findings explain exactly why FN plays roles in suppressing early tumor growth and progression but promoting late cancer metastasis. Whether FN reexpression is a cause or consequence of hampered cell growth, cancer stemness, drug resistance, and cancer metastasis remains to be explored. In addition to effects on tumor cell functionalities, hypoxia greatly impacts TMEs in various respects [179,237]. Unlike the vasculature that is facilitated by the FN deposited in TMEs where nutrients and oxygen are properly provided under normoxia, the neovascular system provoked by HIF-upregulated VEGF under hypoxic conditions is, although abundant, highly abnormal with poor integrity [238,239,240]. Provided that FN deposited in the ECM within TMEs supports a better perfused vasculature and thus promotes rapid tumor growth under normoxia, whether the hypoxia-induced disintegrated vasculature is attributable in part to a FN-deficient ECM remains unclear and is worth investigating. A report has emerged to seemingly oppose the possible regulatory role of FN matrix within TMEs [241]. It claims that, in all conditions tested, the nearly complete absence of FN makes no difference in vascular density and concludes that FN and its receptor subunits are dispensable for tumor angiogenesis [241]. It is not surprising that no difference can be observed with respect to the vascular density in tumor tissues in the presence or absence of ECM FN, as an alteration of vascular growth factors, e.g., VEGF, which are essential for neo-vasculization, has not been evidence. However, the report has not examined whether the integrity of the vasculature within FN-depleted tumor tissues is altered. According to the presented results, the vasculature seems more fragmented and the lengths of visible vessels in the images provided are shorter in the FN-depleted tumor tissues [241], somewhat implicating an abnormality during tumor angiogenesis.

Matrix stiffness in TMEs mechanistically is another mechanical cause for hypoxia-induced tumor metastasis [242]. Matrix stiffness can be made stiffer upon cross-linkages among collagen fibers by LOX, one of products secreted by tumor cells that are under hypoxic conditions [243,244,245]. Matrix stiffness driven by LOX is indeed required for hypoxia-induced tumor metastasis [246]. Interestingly, it has been reported that vigorously growing tumor cells that are deficient in FN synthesis under normoxia in a FN-enriched TMEs are stiffer than adjacent normal cells that capable of expressing FN [214,247]. On the contrary, when tumor cells become more metastatic under chronic hypoxic conditions with a stiffer matrix in TMEs, they turn out to be softer as compared to the less metastatic counterpart tumor cells [214]. The fact that tumor cells and ECM are stiffer in the absence of FN and softer in the presence of FN reflects the possibility that FN plays a role in decreasing the rigidities of ECM in the TMEs and tumor cells to impact tumor progression and metastasis [2,77,83]. The reduced rigidity is likely because of the typical FN characteristics where the conformation is highly changeable and elastic and capable of softening the structurally stiffer and collagenous matrix once interwound with collagen fibers [248,249]. Importantly, it has been well documented that, the increased matrix rigidity induced by hypoxia due to LOX-mediated collagen fiber cross-linkage also contributes to aberrant neovasculization with an abnormal hierarchical arrangement of vascular structures that further promotes tumor metastasis [250,251]. These findings substantially support that a less rigid FN-enriched matrix within TMEs under normoxia promotes the proliferation of primary tumor cells but limits their capacity of metastasis until the matrix within hypoxic environments depletes FN and becomes stiffened. Abundant evidence indicates that CAFs and macrophages are able to change their phenotypes upon hypoxic stimulation to foster a stiffer matrix which in turn aggravating neovascular abnormality and facilitating the subsequent distant organ metastasis [237,252,253]. Indeed, hypoxia-induced CAF and macrophage phenotype alterations actively participate in promoting tumor intravasation, an essential event predisposing tumor cells en route from the circulation to distant organs [136,198,254,255]. Altogether, these findings suggest that hypoxia stands at the crossroad of tumor cell stiffness, tissue stiffness, maturation of tumor angiogenesis, and FN reexpression for tumor suppression and cancer metastasis [205] (Figure 3).

### 4.4. FN Expression in CTCs Facilitates Distant Organ Colonization and Metastasis

CTCs can be found in the very early stage of tumor progression with a large quantity when tumor cells are still deficient in FN expression [256]. These findings are well supported by the fact that CTC alone is insufficient to serve as a precise prognostic marker for cancer metastasis [37,257,258,259], suggesting that a CTC population with metastatic potential is different from other low metastatic CTCs with poorer viability (Figure 3). CTCs make an adventurous journey en route to distant organs with various risky environmental stresses, including anoikis due to a loss of anchorage-dependent cell growth signal stimuli [260], mechanical pressures due to shear stresses and cell deformation within blood vessels of smaller diameter [257], a normal immunosurveillance system competent in destroying CTCs [261], and passing through endothelial barriers to escape the hostile circulation system [262]. A study carefully comparing polarization properties of clinically isolated CTCs and of suspended tumor cell lines exhibits a high degree of similarity [263,264], suggesting that resuspending the attached tumor cell lines may recapitulate characteristics of CTCs derived from cancer patients and is suitable for characterizing CTCs. Indeed, various characteristics of clinically identified metastatic CTCs well coincide with those of resuspended tumor cells [2,75,85,89,265]. Consistently, CTCs may directly be derived from those primary tumor cells who make their way to approach nearby blood vessels to become plakoglobin-mediated CTC clusters that are more resistant to anoikis [266,267,268]. Interestingly, plakoglobin has been demonstrated to maintain FN mRNA stability and increase endogenous FN expression that is required for periFN assembly on primary tumor cells [269], further explaining why FN^high^ CTCs, likely promoting formation of clusters, are more resistant to anoikis and better survive in the circulation. Depletion of plakoglobin drastically reduces FN expression, CTC cluster formation, and distant metastasis [270]. In line with these findings, it has been shown that although CTC clusters are much rarer as compared to single CTCs, the metastatic potential appears to be significantly higher, confirming that CTC clusters survive better within the circulation prior to distant colonization and metastatic growth [266,271]. Additionally, formation of tumor cell–platelet microaggregates, resulting in physical shielding, has been demonstrated to protect CTCs from mechanical stress-induced cell deformation and cell death [37,272,273]. Cumulative reports have provided evidence to ascribe resistance of CTCs to mechanical deformation to CTC-platelet microaggregates in which adhesion between platelet integrin, e.g., αIIbβ3 (glycoprotein IIb/IIIa), and tumor cell integrin ligands, e.g., periFN [274,275], is triggered [266,268,271]. Conversely, since TGF-β1 has been shown to be a major regulator for endogenous FN synthesis [276,277], platelet microaggregates may prompt secretion of TGF-β1 by platelets to further sustain E–M plasticity of CTCs, likely facilitating periFN assembly on tumor cells to make aggregation of CTCs with platelets stronger, rendering more resistance of CTCs to mechanical deformation in the circulation [278]. Another way metastatic CTCs utilize to fight against shear stress- and cell deformation-induced apoptosis is the formation of EMT-facilitated microtentacles [36], particular cytoskeletal structures composed of Glu-tubulin to generate stabilized detyrosinated microtubules [279]. These structures can particularly be enriched by a well-known mesenchymal marker vimentin [280]. Importantly, endogenous FN expression in tumor cells has been found to be promoted by upregulation of vimentin during E–M plastic processes [281,282], suggesting again that elevated endogenous FN expression in metastatic CTCs results in better periFN assembly to sustain stability of microtentacles, which not only protect CTCs from mechanical stresses existing in the circulation but also pave ways for CTCs to colonize distant organs. This conclusion is well supported by the findings that vimentin inhibition with withaferin A abolishes binding of FN to integrin α5β1, one of cell surface receptors mediating periFN assembly [283]. Further supporting evidence is that the ability of vimentin in controlling integrin recycling depends on the phosphorylation mediated by PKCε [284], which has been revealed to promote periFN assembly on blood-borne breast cancer cells and lung metastasis [265]. In line with these findings, it has been demonstrated that vimentin expression can serve as a prognostic biomarker for the metastases in non-small cell lung carcinoma patients [285].

Among various peripheral leukocytes responsible for the active immunosurveillance in the circulation, natural killer (NK) cells are majorly involved in anti-tumor cytotoxicity as exhibited in humans and animal models with defective NK cell function [286]. CTCs with E–M plasticity are able to secrete TGFβ, which serves as a potent inhibitor of NK cytotoxicity against tumor cells by downregulating activating receptor NKG2D expression on NK cells [286]. Since FN is a major E–M plasticity marker, it is conceivable that periFN may mediates the TGFβ-dependent immune escape of CTCs from NK cytotoxicity. As it is a requirement for NK cells to directly interact with CTCs prior to effective tumor lysis, a physical shielding may help CTCs escape NK immunosurveillance [37]. Indeed, it has been shown that formation of CTC-platelet microaggregates efficiently prevents NK cytotoxicity [287,288]. Alternatively, activated platelets in these microaggregates can expose NK cell inhibitory ligands [289]. Since FN expression in CTCs is critically involved in the formation of CTC–platelet microaggregates, the possibility that periFN on CTCs plays an important role in immune evasion for NK insulting is further substantiated. In contrast to NK cells, macrophages and neutrophils are among innate immune cells playing an opposite role to protect CTCs from anoikis and mechanical deformation-induced cell death [290,291,292]. Interestingly, pro-metastatic neutrophils may alternatively impact immune escape via suppressing cytotoxic CD8+ T cells and NK cells [293,294,295]. Recently, programmed death-ligand 1 (PD-L1), the ligand for the cytotoxic T lymphocyte immune checkpoint receptor programmed death 1 (PD-1) during immunosuppression processes within primary tumor tissues, has also been detected on the cell surfaces of CTCs derived from cancer patients [296,297], implying that T cell-mediated adaptive immunosurveillance is also in effect in the circulation and CTCs may escape such immune assault by the engagement of PD-1/PD-L1 immune checkpoint [36]. Indeed, miR200/ZEB1 axis-induced EMT pathways also regulate PD-L1 expression in CTC [232,298] and FN expression is evidenced when miR200 is overexpressed or ZEB1 mRNA is stabilized [299,300,301], indicating the possibility that periFN on CTCs exerts PD-1/PD-L1-mediated immune checkpoint to escape T-cell-mediated cytotoxicity.

To accomplish metastatic growth in distant organs, several requisite steps for CTCs to extravasate the blood stream, including attachment to endothelia of distant organs, endothelial retraction, and transendothelial migration [37]. Importantly, periFN assembly on CTCs of various cancer types has been shown in in vivo tumor colonization assays to mediate cancer metastasis in the lungs via binding to lung endothelial adhesion receptor dipeptidyl peptidase IV (DPP IV; also named CD26) [1,2,4,6,75,76]. Furthermore, employing a novel in vivo imaging experimental designing in which endothelia-attaching CTCs can be visualized within the vasculature of lung tissues, it has been demonstrated that periFN is a mandatory tumor cell surface ligand responsible for DPP IV-binding prior to extravasation in the lung tissues [302]. Whereas numerous studies have been dedicated to exploring molecular mechanism underlying periFN assembly on adherent cells, how CTCs assemble periFN in suspension is just about to be understood. Protein kinase Cε (PKCε) has been shown to regulate periFN assembly on suspended rat breast tumor cells when it is upregulated and phosphorylated, leading to a rapid translocation of PKCε from cytosol to plasma membrane [265]. Specific PKCε inhibitors block PKCε phosphorylation and expression followed by a suppression of plasma membrane translocation and periFN assembly, substantially supporting the essential role of PKCε in periFN assembly. As expected, tumor metastasis in the lungs can be drastically abolished [265]. A comprehensive secretomic study and quantitative analyses reveal that 68 out of 660 secreted proteins can be identified to be differentially expressed in FN^high^ and FN^low^ human lung tumor cells [2]. Among 68 proteins, A1AT as a serine protease inhibitor has further been identified to be highly secreted in FN^high^ cell line CL1-5, facilitating CL1-5 metastasis in the lungs. Depletion of A1AT from CL1-5 cells significantly reduces periFN assembly and thus lung metastasis, suggesting that periFN assembly can also be modulated on suspended tumor cell surfaces via A1AT [2]. Consistently, a DPP IV-derived polypeptide harboring the FN-binding site specifically blocks A1AT-promoted lung colonization of CL1-5 with a high level of periFN assembly, confirming that the specific adhesion between periFN on CTCs and DPP IV on lung endothelia readily promotes tumor metastasis in the lungs [2]. This notion is further substantiated by the fact that Lung metastasis of FN^high^ rat breast cancer cells can be effectively blocked by FN-derived polypeptides harboring the consensus DPP IV-binding motif [6]. Moreover, thanks to a natural phytochemical pterostilbene that potently reduces periFN assembly on suspended lung cancer cells and their metastasis in the lungs, the AKT-ERK signaling axis has separately been identified in regulating periFN assembly on CTCs [4]. It is worthwhile to test the possibility that PKCε AKT-ERK axis, and A1AT are causally connected to concertedly regulate periFN assembly on CTCs. Upon attachment of CTCs to endothelia, disruption of endothelial integrity and retraction of endothelial cells appear to be essential in facilitating metastatic growth. CTC-secreted angiopoietin-like 4 (ANGPTL4) or its C-terminal fragment are competent in antagonizing vascular endothelial cell-cell junctions to facilitate extravasation and metastasis of tumor cells due to direct interaction with adhesion molecules, e.g., a FN receptor α5β1, involved in cell junctions [303,304]. Interestingly, ANGPTL4 has been deemed a regulator of endogenous FN synthesis in tumor cells likely through an autocrine or paracrine manner [305]. Conceptually, ANGPTL4 may alternatively induce endothelial retraction by promoting periFN assembly on CTCs that leads to an enhancement of tumor-endothelial adhesion via periFN-DPP IV binding. This possibility is worth further investigating. Moreover, it has been reported that ablating VCAM-1-mediated actin cytoskeleton organization causes endothelial junction opening [306], and endothelial cell death in a form of necroptosis generates gaps in endothelia [307], leading to an endothelial barrier opening [308]. Whether these endothelial integrity-disrupting events are directly induced by endothelial DPP IV upon stimulation with cancerous periFN-binding remains to be examined. Importantly, cumulative evidence indicates that impairment of several ligand–receptor interactions between CTCs and endothelia are required for promoting cancer metastasis through enhancing transendothelial migration without affecting cell-cell adhesions between them [37,309,310,311,312]. It is worthwhile to corroborate whether the impairment of these homophilic and heterophilic interactions can be initiated by the adhesion between cancerous periFN and endothelial DPP IV to facilitate transendothelial migration of CTCs and promote cancer metastasis in distant organs.

### 4.5. Malignant Tumor Cells in the Primary Tissues Early Establish PMN in Distant Tissues and Disseminated Tumor Cells Continue to Evolve, Rendering Macrometastatic Outgrowth

To complete the entire process of metastatic growth in distant organs, extravasated FN^high^ tumor cells must conquer growth disadvantages conveyed by autonomous and nonautonomous tumor suppressive stresses [313,314]. These disadvantages ultimately lead to tumor dormancy and formation of micrometastasis until the unfavored factors are cleared [315,316]. Cancerous FN may exert tumor suppressive functions as aforementioned to halt cell proliferation, migration, invasion, and angiogenesis [51,52,54,56] once CTCs arrive at distant organs and reside in the parenchymal tissues, reminiscence of scenario in the primary tumor tissues. Imaginably, new and different locally imposed innate and adaptive immunosurveillances from those in the primary tumor tissues can form huge microenvironmental pressures to further suppress metastatic outgrowth [313,314], turning tumor cells into senescence-like dormancy in the micrometastatic foci within which dormant tumor cells keep evolving [316]. Such tumor dormancy in micrometastasis can last for several years until tumor cells successfully evolve to outgrow into macrometastasis with the growth-advantageous supporting from tumor cell-driven favorable pro-tumor microenvironments [317] (Figure 2 and Figure 3). Since FN is a major EMT marker expressed by tumor cells and can be upregulated by transcription factors, e.g., Twist1 or Snail1, the role of EMT in formation of FN^high^ tumor dormancy at distant sites has been evidenced [259,318]. It has been presented that disseminated tumor cells (DTCs) that enter dormancy and form micrometastasis highly express Twist1 or Snail1 [319,320], suggesting that EMT contributes to the initial colonization and formation of dormancy. Consistently, an early subpopulation of DTCs in the Her2-driven mouse breast cancer model also exhibits a Twist^high^ E-cadherin^low^ phenotype [321]. Consistently, tumor cells outgrown in the macrometastatic foci often display pro-epithelial instead of pro-mesenchymal phenotypes, seemingly challenging the requirement of E–M plasticity in metastatic establishment [322,323,324,325]. Nevertheless, it has been demonstrated that, by tracking the primary tissue-inoculating tumor cells with pro-mesenchymal phenotypes, outgrown DTCs in the macrometastatic foci exhibit pro-epithelial phenotypes, indicative of an apparent reversion of MET from EMT phenotypes [326]. These findings well explain why evolved pro-epithelial phenotypes of DTCs are displayed in the macrometastatic foci that initially originate from extravasated CTCs with pro-mesenchymal phenotypes [319,320] and outgrow from the dormant micrometastasis due to continued evolution [327], a scenario reminiscent of early tumor progression where tumor cells vigorously proliferate, migrate, invade surrounding ECMs, promote angiogenesis when FN expression is downregulated in a pro-epithelial phenotype as aforementioned [54,55,56]. Indeed, suppression of tissue factors (TFs) that are competent of driving E–M plasticity is a mandatory step to promote macrometastatic outgrowth at distant organs after the initial steps of CTC colonization that require pro-mesenchymal phenotypes [328,329]. Similarly, downregulation of EMT inducers Prrx1, ID1, or Snail1, transcription factors for FN expression [330,331,332], effectively promotes macrometastasis [330,333,334]. The role of FN expression in DTCs in metastatic dormancy is further substantiated by the findings in which stem-like prostate tumor cells were used to perform an in vivo selection and the indolent cell line preserved the dormant state when implanted into tibial bones, whereas aggressive cell line proliferated rapidly in bones. Secreted protein acidic and rich in cysteine (SPARC), known as a promoter for periFN assembly, was identified to be highly expressed by the indolent cells [335]. Knocking down SPARC expression significantly waken tumor dormancy and drastically lowered bone metastasis-free survival of tumor-bearing mice, suggesting SPARC plays a central role in maintaining tumor dormancy [335]. Conceivably, the effect of SPARC on tumor dormancy may be due to the elevated level of periFN assembly, which is worth further researching.

Another intriguing question is whether tumor dormancy in FN^high^ DTCs at distant organs can be reversed by the M–E phenotype without affecting the expression level of FN in DTCs. To answer this question, we have focused on two important mouse mammary adenocarcinoma cell lines, 4T1 and 67NR, which were isolated spontaneous mammary tumor in a BALB/cfC3H mouse [336]. Interestingly, it has been demonstrated that 4T1 cells are highly metastatic but exhibit pro-epithelial phenotypes, e.g., upregulation of E-cadherin and tight junction protein ZO-1. Conversely, 67NR cells possess pro-mesenchymal phenotypes, e.g., higher levels of N-cadherin and vimentin but without E-cadherin and do not form macrometastasis foci in the lungs at the time 4T1 cells already generate lots of metastatic nodules when inoculated in fat pad or intravenously injected into syngeneic mice [337,338]. However, this work does not provide evidence as to how these two cell lines express endogenous FN and assemble periFN. In our unpublished results, we found that both cells were competent in expressing FN but there were no differences in levels of FN expression and periFN assembly between two cell lines. These findings are strongly supported by the gene profiling as performed with cDNA microarrays where FN is absent among 53 upregulated and 74 downregulated genes in 4T1 cells as compared to 67NR cells [338]. The fact that both 4T1 and 67NR cells are highly expressing FN and assembling periFN [unpublished data], but only 4T1 cells characterized with pro-epithelial phenotypes are competent in forming macrometastasis foci in the lungs [338], implying that, although FN^high^ 67NR cells are capable of attaching lung endothelia and form micrometastasis in the lung parenchyma, their pro-mesenchymal phenotypes render tumor dormancy and hamper macrometastatic outgrowth. Indeed, employing a lung colonization assay for circulating tumor cell visualization in lung tissues [302], we found that the abilities of both cell lines to colonize the lungs were about the same, but tail vein-injected 67NR cells formed tumor nodules in the lungs in about 2 months, which was much slower than 4T1 cells did in as early as the 10th day after tumor injection [unpublished data]. These findings reason why the FN^high^ phenotype of the metastatic tumor cells, including 4T1 cells, can repeatedly be isolated from macrometastatic foci in the metastatic tissues of mice intravenously or orthotopically inoculated with tumor cells [77,339,340]. These findings also confirm that the FN expression and periFN pertinent to E–M phenotypes are required for FN^high^ CTC colonization but render tumor dormancy in distant organs and M–E phenotypes drives FN^high^ DTCs outgrowth from dormancy and set forth an intriguing possibility that functionalities of individual molecules involved in E–M and M–E plasticity and phenotypical changes between epithelial and mesenchymal morphologies can be uncoupled. This possibility can be echoed by the findings in which reversion pro-mesenchymal into pro-epithelial phenotypes awakes DTCs from dormancy and promotes metastatic outgrowth and loss of E-cadherin, one of pro-epithelial biomarker, unambiguously hampers cancer cell growth and survival, reduces numbers of CTCs and extravasated DTCs in distant organs, and lowers metastatic outgrowth regardless of the E/M phenotype state [341,342]. Another example is that binding of breast cancer cells to endothelial E-selectin can promote bone-metastasis by triggering non-canonical M–E reversion concomitantly with Wnt-upregulated FN [343]. In addition to autonomous evolution that promotes macrometastatic tumor outgrowth, local microenvironments are deemed important for transition of M–E phenotypes in the distant organs [318]. A non-autonomous effect exerted by FN^high^ DTCs on bone marrow-derived cells (BMDCs) has been ascribed to DTC-secreted SPARC that drives expression of BMP7 in BMDCs to maintain tumor dormancy by inducing senescence in micrometastatic cancer cells [335]. These findings unveil an essential role of SPARC within TMEs of the bone in sustaining prostate tumor dormancy and suggest that SPARC inactivation may lead to M–E reversion and evasion of BMDC-driven tumor dormancy. Whether a high level of FN expression can be maintained during the M–E reversion by inactivating SPARC remains to be clarified. CAF is another cell type to be activated and recruited by AXL^high^ mesenchymal DTCs in the micrometastatic foci, followed by an activated CAF-stimulated M–E phenotype reversion and outgrowth of DTCs [344]. AXL has been recognized as a strong E–M plasticity and cancer stemness inducer, resulting in apparent upregulation of pro-mesenchymal markers including FN [345]. Interestingly, when M–E phenotype of AXL^high^ mesenchymal DTCs was reversed upon stimulation by activated CAFs, only AXL, Twist1, and vimentin were downregulated, but FN was not examined [344]. These findings may imply that during M–E phenotype reversion in FN^high^ DTCs, FN expression is maintained regardless of alterations of other E/M marker expression as aforementioned [77,339].

It is now well recognized that primary tumors also contribute metastatic-promoting factors in a systemic manner to establish favorable microenvironments, so-called PMN, at distant organs prior to the arrival of DTCs [346]. Ample evidence indicates that the recruitment of CD11b+ BMDCs plays an important role in the establishment of PMN [40,41,42,43,44]. Interestingly, FN deposited in the PMN has been deemed as an essential player in promoting macrometastatic outgrowth [40,346]. Predisposed primary tumor cells with metastatic potency may secrete, at the primary sites, soluble chemoattractants [41], or exosomes harboring stimulatory factors to upregulate FN expression in, most likely, locally recruited TAMs or CAFs, followed by ECM deposition and remodeling [40,346]. Subsequently, the VEGFR1^+^ BMDCs are mobilized by those chemoattractants to arrive at FN-enriched ECM and attach to the deposited FN via surface-expressed integrin α4β1 [40]. Consistently, distinct integrins expressed on the surfaces of primary tumor-derived exosomes can determine organ-specific cancer metastasis by binding to their corresponding matrix proteins in the PMNs [44,347]. Such microenvironmental cues are highly reminiscent of those in the FN-enriched primary tissues where tumor cells vigorously grow and progress. These findings reason why the FN^high^ dormant DTCs need to be reversed into pro-epithelial phenotypes in that pro-mesenchymal phenotypes with a high level of FN expression, like those in the primary tumor tissues, disfavor outgrowth of DTCs. By clinical observations, it takes an awfully long latent period of dormant state from extravasation of CTCs into distant organs and become micrometastatic DTCs to macrometastatic outgrowth during late stage of metastatic progression [259]. However, distant metastatic recurrences are often rapidly developed in those patients who receive surgical removal of their primary tumors [259,316]. Altogether, these observations raise a fascinating possibility that PMNs and metastatic niche at least share certain contributors to facilitate macrometastatic outgrowth. Indeed, in vivo experiments have been designed to explore such possibility. The results decipher that systemic inflammatory response caused by surgical operations unambiguously promotes macrometastatic outgrowth of DTCs which are otherwise maintained and controlled by local innate as well as adaptive immunosurveillances in a dormant state in distant organs as corroborated by a perioperative anti-inflammation treatment that drastically suppresses macrometastatic outgrowth [316]. It appears to be clear that pro-tumor inflammatory cytokines and chemokines are important characteristics shared by the TMEs of primary tissues, metastatic niche, and PMN to potently result in accumulation and polymerization of FN and promote tumor cell outgrowth [41]. It would be interesting to investigate whether these FN-enriched niches can force further tumor evolution by a M–E phenotype reversion [344]. Our unpublished observations resulting from two highly metastatic cell lines FN^high^ MDA-MB-231 and FN^low^ MDA-MB-435 only begin to shed light on these causal relationships [348]. Expectedly, when intravenously injected, significantly more FN^high^ MDA-MB-231 cells than FN^low^ MDA-MB-435 cells [unpublished data] [348] formed tumor nodules in the female athymic nude mouse lungs. Moreover, MDA-MB-435 cells, but not MDA-MB-231, could rapidly grow in mouse mammary fatpads [unpublished data], consistent with the tumor suppressive role of FN expression in early tumor progression. Surprisingly, in spontaneous metastasis assays, only MDA-MB-435 cells, but not MDA-MB-231 cells, entered the circulation and extravasated as DTCs to form tumor nodules in the mouse lungs [348] [unpublished data]. These findings imply that FN^low^ MDA-MB-435 cells with pro-epithelial phenotypes are competent in establishing pro-tumor FN-enriched TMEs to nourish pro-epithelial FN^low^ tumor cells, rendering tumor outgrowth in the mammary fatpads. These vigorously growing FN^low^ cells, but not the slow-growing FN^high^ cells, may secrete cytokines, chemokines, and exosomes to mobilize BMDCs to the future metastatic sites where the FN-enriched PMNs can then be established, waiting for the future arrival and outgrowth of MDA-MB-435 DTCs [41]. Uncoupling of M–E reversion from FN expression explains why FN^high^ tumor cells can repeatedly be isolated from macrometastatic foci in distant organs [77]. Such phenomenon also explains well why DTCs outgrown in the first metastatic site can reenter the circulation and colonize the second metastatic site [349,350], likely through reversible E–M and M–E plasticity without affecting periFN assembly on their cell surfaces. Aforementioned interchanges of various phenotypes in tumor cells, stromal cells, and stromal FN during the whole processes of tumor genetic/epigenetic evolution and progression where hypoxia and PMN are critically at the crossroads are illustrated in Figure 4.

## 5. Future Perspectives for FN-Targeting Therapeutic Strategies against Cancer

**Figure 5:**
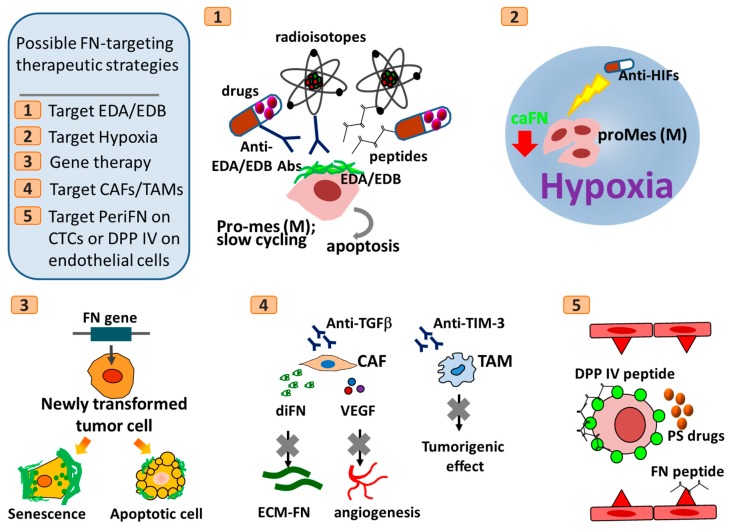
Hypothetic scheme of possible FN-targeting therapeutic strategies. Abbreviations: CAFs, cancer-associated fibroblasts; TAMs, tumor-associated macrophages; TIM-3, T cell immunoglobulin and mucin domain-containing molecule-3; HIFs, hypoxia-induced factors; CTCs, circulating tumor cells; PS, pterostilbene; periFN, pericellular fibronectin; EDA, extra domain A of fibronectin; EDB, extra domain B of fibronectin; Abs, antibodies; diFN, dimeric form of fibronectin; caFN, cancerous fibronectin; ECM-FN, fibronectin deposited in ECM; VEGF, vascular endothelial growth factor; DPP IV, dipeptidyl peptidase IV. All other cartoon characters and abbreviations are referred to Figure 2, Figure 3 and Figure 4.

Since how cancerous FN and ECM FN participate in tumor progression remains obscure and paradoxical, FN-based cancer therapies have so far only been focused on the functions of drug delivery. For example, EDA- and EDB-containing oncofetal variants have often been utilized for that purpose [3,5,351,352]. Despite lots of intriguing phenomena and underlying molecular mechanisms remain unresolved and urgently need to be fully deciphered, cellular stresses-triggered loss of FN expression, hypoxia-driven FN reexpression and the E–M/M–E phenotypic reversion which can be uncoupled from FN functionality seem to be major determinants dictating the tumor suppressive and metastatic-promoting roles of FN in temporal and spatial manners during tumor progression and can serve as targets for developing cancer therapeutic strategies (Figure 5). In the very early stage of tumor progression, FN is downregulated to help tumor cells ameliorate their oncogene- or loss of TSG-induced endogenous ER stresses and evade senescent limitation of cell cycle progression. Therefore, for tumor patients in a very early clinical stage, in addition to the traditional surgery, chemotherapies, and radiotherapies or recently prevailing immune checkpoint blockade therapies [353,354,355], therapeutic strategies can be formed by inducing overexpression of FN in newly transformed tumor cells and force tumor cells into senescent state or cell cycle arrest followed by cell apoptosis, if FN is proved to be a regulator for senescent induction by enhancing ER stresses. Alternatively, tumor senescence and subsequently apoptosis can be induced circumventing alteration of FN expression level [356,357]. However, one need to use caution that these FN-based therapeutic strategies should not be practiced once CTCs are identified from tumor patients’ blood samples in that elevated levels of endogenous and thus periFN assembly in tumor cells may make FN^high^ CTCs colonize more and form more metastatic tumor nodules in distant organs, unless there are strategies to reverse FN^high^ CTCs back to pro-epithelial phenotypes that tend to render CTCs suffer more from anoikis and mechanical stresses and injuries.

At the same stage of tumor patients, FN deposition in ECM of TMEs exacerbates tumor growth and subsequent progression. Therefore, approaches targeting such matrix-depositing FN may be ideal for effectively slowing down the tumor progression. FN secretion and deposition may due in part to tumor-infiltrating pro-tumor stromal cells including CAFs and TAMs [358]. Ablation of these tumor-infiltrating cells could be an anti-tumor strategy [359,360,361]. For example, Fresolimumab, a monoclonal antibody against TGF-β to block FN secretion and VEGF secretion from activated CAFs, has been used in clinical trials for melanoma, renal cell carcinoma, and mesothelioma [362]. Moreover, TAMs can be activated by T cell immunoglobulin and mucin domain-containing molecule-3 (TIM-3) and, in an in vivo kidney cancer model, blocking antibodies developed against TIM-3 has been shown to reduce its tumorigenic effects [363].

In later stages of tumor progression, tumor cells with the metastatic potential begin to intravasate into the circulation and become FN^high^ CTCs prior to extravasation in distant organs as DTCs. Blockade of the attachment of FN^high^ CTCs to DPP IV-expressing endothelia can be an ideal strategy to prevent tumor metastasis and improve patients’ prognosis. Indeed, in experimental metastasis assays, polypeptides derived either from FN harboring DPP IV-binding sites [6] or from DPP IV harboring FN-binding sites [1,2] have been identified and produced to significantly suppress rat mammary adenocarcinoma tumor metastasis in the lungs [75]. More importantly, how to concomitantly prevent tumor metastasis in distant organs and suppress distant tumor outgrowth to prolong patients’ survival becomes the most urgent anti-tumor therapeutic strategies. Screening stilbenoids, pterostilbene (PS), a well-known phytochemical capable of triggering strong apoptosis in attached tumor cells, has been found to possess the best potency in suppressing periFN assembly on suspended tumor cells existing in the circulation as a form of CTCs [4]. Intriguingly, by oral gavage, PS was able to prevent intravenously injected mouse Lewis lung cancer cells from entering the lungs and also significantly inhibited the outgrowth of already lung-colonized DTCs by exerting the apoptotic effect on the extravasated CTCs [4].

In most cases, tumor patients receive either tradition chemo/radiotherapies or targeted anti-tumor therapies. As aforementioned, tumor patients often develop resistance to these anti-cancer drugs, driving tumor evolution, malignant progression, intravasation of tumor cells to become CTCs, and metastatic recurrence simultaneously with the upregulation of endogenous FN. Although therapy-elevated FN expression in resistant tumor cells slow down the tumor growth, the blood-borne FN^high^ CTCs become highly metastatic and capable of colonizing distant organs. The prevailing therapeutic strategies are to switch to a second line of tumor-killing drugs or combinatory therapies including immune checkpoint blockade, which may cause further genetic or epigenetic evolutions and eventually lead to another drug resistance. These problems may be avoided by finding a drug that by itself has no cytotoxic effect on tumor cells but is able to sensitize the resistant tumor cells to the original drugs. Efforts can be exerted to screening such drug from either small molecule library or a series of less harmful natural phytochemicals. By combining such drug with periFN/DPP IV binding blockade treatments, the drug-resistant tumor cells can be re-sensitized and controlled in the primary sites and FN^high^ CTCs can simultaneously prevented from entering distant sites.

## 6. Conclusions

In summary, after extensively reviewing the bulk literature regarding the roles of FN in cancer progression, we provided intriguing possibilities that reasonably reconcile the seemingly paradoxical roles FN plays. We hypothesized that cancerous FN and FN mactrices in TMEs can coordinately regulate tumor transformation and malignant progression in temporal and spatial manners. Upon accomplished experimental proof of concept, FN can be carefully targeted at the right location and in the right time. We finally provided a few FN-based therapeutic strategies as future perspectives. Hopefully, this review article can attract more cancer researchers to be delved into concerted efforts in unveiling and validating what had been proposed here.

## References

[1] Abdel-Ghany M., Cheng H., Levine R.A., Pauli B.U. (1998). Truncated dipeptidyl peptidase IV is a potent anti-adhesion and anti-metastasis peptide for rat breast cancer cells. Invasion Metastasis.

[2] Chang Y.H., Lee S.H., Liao I.C., Huang S.H., Cheng H.C., Liao P.C. (2012). Secretomic analysis identifies alpha-1 antitrypsin (A1AT) as a required protein in cancer cell migration, invasion, and pericellular fibronectin assembly for facilitating lung colonization of lung adenocarcinoma cells. Mol. Cell. Proteom..

[3] Berndorff D., Borkowski S., Sieger S., Rother A., Friebe M., Viti F., Hilger C.S., Cyr J.E., Dinkelborg L.M. (2005). Radioimmunotherapy of solid tumors by targeting extra domain B fibronectin: Identification of the best-suited radioimmunoconjugate. Clin. Cancer Res..

[4] Wang Y.J., Lin J.F., Cheng L.H., Chang W.T., Kao Y.H., Chang M.M., Wang B.J., Cheng H.C. (2017). Pterostilbene prevents AKT-ERK axis-mediated polymerization of surface fibronectin on suspended lung cancer cells independently of apoptosis and suppresses metastasis. J. Hematol. Oncol..

[5] Moschetta M., Pretto F., Berndt A., Galler K., Richter P., Bassi A., Oliva P., Micotti E., Valbusa G., Schwager K. (2012). Paclitaxel enhances therapeutic efficacy of the F8-IL2 immunocytokine to EDA-fibronectin-positive metastatic human melanoma xenografts. Cancer Res..

[6] Cheng H.C., Abdel-Ghany M., Pauli B.U. (2003). A novel consensus motif in fibronectin mediates dipeptidyl peptidase IV adhesion and metastasis. J. Biol. Chem..

[7] Beier U.H., Holtmeier C., Weise J.B., Gorogh T. (2007). Fibronectin suppression in head and neck cancers, inflammatory tissues and the molecular mechanisms potentially involved. Int. J. Oncol..

[8] Akiyama S.K., Olden K., Yamada K.M. (1995). Fibronectin and integrins in invasion and metastasis. Cancer Metastasis Rev..

[9] Taylor G.A., Jeffers M., Webb C.P., Koo H.M., Anver M., Sekiguchi K., Vande Woude G.F. (1998). Decreased fibronectin expression in Met/HGF-mediated tumorigenesis. Oncogene.

[10] Santiago-Medina M., Yang J. (2016). MENA Promotes Tumor-Intrinsic Metastasis through ECM Remodeling and Haptotaxis. Cancer Discov..

[11] Topalovski M., Brekken R.A. (2016). Matrix control of pancreatic cancer: New insights into fibronectin signaling. Cancer Lett..

[12] Han S.W., Roman J. (2006). Fibronectin induces cell proliferation and inhibits apoptosis in human bronchial epithelial cells: Pro-oncogenic effects mediated by PI3-kinase and NF-kappa B. Oncogene.

[13] Erdogan B., Ao M., White L.M., Means A.L., Brewer B.M., Yang L., Washington M.K., Shi C., Franco O.E., Weaver A.M. (2017). Cancer-associated fibroblasts promote directional cancer cell migration by aligning fibronectin. J. Cell Biol..

[14] Attieh Y., Clark A.G., Grass C., Richon S., Pocard M., Mariani P., Elkhatib N., Betz T., Gurchenkov B., Vignjevic D.M. (2017). Cancer-associated fibroblasts lead tumor invasion through integrin-beta3-dependent fibronectin assembly. J. Cell Biol..

[15] Kuonen F., Surbeck I., Sarin K.Y., Dontenwill M., Ruegg C., Gilliet M., Oro A.E., Gaide O. (2018). TGFbeta, Fibronectin and Integrin alpha5beta1 Promote Invasion in Basal Cell Carcinoma. J. Investig. Dermatol..

[16] Christensen L., Nielsen M., Andersen J., Clemmensen I. (1988). Stromal fibronectin staining pattern and metastasizing ability of human breast carcinoma. Cancer Res..

[17] Takei H., Iino Y., Horiguchi J., Yokoe T. (1995). Immunohistochemical fibronectin staining pattern and prognosis in invasive breast carcinoma. Oncology.

[18] Jagirdar J., Ishak K.G., Colombo M., Brambilla C., Paronetto F. (1985). Fibronectin patterns in hepatocellular carcinoma and its clinical significance. Cancer.

[19] Stenman S., Vaheri A. (1981). Fibronectin in human solid tumors. Int. J. Cancer.

[20] Knudson A.G. (1996). Hereditary cancer: Two hits revisited. J. Cancer Res. Clin. Oncol..

[21] Knudson A.G. (1971). Mutation and cancer: Statistical study of retinoblastoma. Proc. Natl. Acad. Sci. USA.

[22] Weinhold N., Ashby C., Rasche L., Chavan S.S., Stein C., Stephens O.W., Tytarenko R., Bauer M.A., Meissner T., Deshpande S. (2016). Clonal selection and double-hit events involving tumor suppressor genes underlie relapse in myeloma. Blood.

[23] Williams M.J., Werner B., Barnes C.P., Graham T.A., Sottoriva A. (2016). Identification of neutral tumor evolution across cancer types. Nat. Genet..

[24] Zhao X., Gao S., Wu Z., Kajigaya S., Feng X., Liu Q., Townsley D.M., Cooper J., Chen J., Keyvanfar K. (2017). Single-cell RNA-seq reveals a distinct transcriptome signature of aneuploid hematopoietic cells. Blood.

[25] Fitzgerald D.M., Hastings P.J., Rosenberg S.M. (2017). Stress-Induced Mutagenesis: Implications in Cancer and Drug Resistance. Annu. Rev. Cancer Biol..

[26] Yu Y.R., Ho P.C. (2019). Sculpting tumor microenvironment with immune system: From immunometabolism to immunoediting. Clin. Exp. Immunol..

[27] O’Donnell J.S., Teng M.W.L., Smyth M.J. (2019). Cancer immunoediting and resistance to T cell-based immunotherapy. Nat. Rev. Clin. Oncol..

[28] Rankin E.B., Giaccia A.J. (2016). Hypoxic control of metastasis. Science.

[29] Brown J.M., Giaccia A.J. (1998). The unique physiology of solid tumors: Opportunities (and problems) for cancer therapy. Cancer Res..

[30] Chan C.Y., Yuen V.W., Wong C.C. (2019). Hypoxia and the Metastatic Niche. Adv. Exp. Med. Biol..

[31] Peinado H., Zhang H., Matei I.R., Costa-Silva B., Hoshino A., Rodrigues G., Psaila B., Kaplan R.N., Bromberg J.F., Kang Y. (2017). Pre-metastatic niches: Organ-specific homes for metastases. Nat. Rev. Cancer.

[32] Janker L., Mayer R.L., Bileck A., Kreutz D., Mader J.C., Utpatel K., Heudobler D., Agis H., Gerner C., Slany A. (2019). Metabolic, Anti-apoptotic and Immune Evasion Strategies of Primary Human Myeloma Cells Indicate Adaptations to Hypoxia. Mol. Cell. Proteom..

[33] Pantel K., Speicher M.R. (2016). The biology of circulating tumor cells. Oncogene.

[34] Joosse S.A., Gorges T.M., Pantel K. (2015). Biology, detection, and clinical implications of circulating tumor cells. Embo. Mol. Med..

[35] Micalizzi D.S., Maheswaran S., Haber D.A. (2017). A conduit to metastasis: Circulating tumor cell biology. Genes Dev..

[36] Francart M.E., Lambert J., Vanwynsberghe A.M., Thompson E.W., Bourcy M., Polette M., Gilles C. (2018). Epithelial-mesenchymal plasticity and circulating tumor cells: Travel companions to metastases. Dev. Dyn..

[37] Strilic B., Offermanns S. (2017). Intravascular Survival and Extravasation of Tumor Cells. Cancer Cell.

[38] Yadav A.S., Pandey P.R., Butti R., Radharani N.N.V., Roy S., Bhalara S.R., Gorain M., Kundu G.C., Kumar D. (2018). The Biology and Therapeutic Implications of Tumor Dormancy and Reactivation. Front. Oncol..

[39] Jolly M.K., Ware K.E., Gilja S., Somarelli J.A., Levine H. (2017). EMT and MET: Necessary or permissive for metastasis?. Mol. Oncol..

[40] Kaplan R.N., Riba R.D., Zacharoulis S., Bramley A.H., Vincent L., Costa C., MacDonald D.D., Jin D.K., Shido K., Kerns S.A. (2005). VEGFR1-positive haematopoietic bone marrow progenitors initiate the pre-metastatic niche. Nature.

[41] Hiratsuka S., Watanabe A., Aburatani H., Maru Y. (2006). Tumour-mediated upregulation of chemoattractants and recruitment of myeloid cells predetermines lung metastasis. Nat. Cell Biol..

[42] Deng J., Liu Y., Lee H., Herrmann A., Zhang W., Zhang C., Shen S., Priceman S.J., Kujawski M., Pal S.K. (2012). S1PR1-STAT3 signaling is crucial for myeloid cell colonization at future metastatic sites. Cancer Cell.

[43] Erler J.T., Bennewith K.L., Cox T.R., Lang G., Bird D., Koong A., Le Q.T., Giaccia A.J. (2009). Hypoxia-induced lysyl oxidase is a critical mediator of bone marrow cell recruitment to form the premetastatic niche. Cancer Cell.

[44] Peinado H., Aleckovic M., Lavotshkin S., Matei I., Costa-Silva B., Moreno-Bueno G., Hergueta-Redondo M., Williams C., Garcia-Santos G., Ghajar C. (2012). Melanoma exosomes educate bone marrow progenitor cells toward a pro-metastatic phenotype through MET. Nat. Med..

[45] Hynes R.O. (1973). Alteration of cell-surface proteins by viral transformation and by proteolysis. Proc. Natl. Acad. Sci. USA.

[46] Hynes R. (1990). Fibronectins.

[47] Leahy D.J., Aukhil I., Erickson H.P. (1996). 2.0 A crystal structure of a four-domain segment of human fibronectin encompassing the RGD loop and synergy region. Cell.

[48] Schwarzbauer J.E. (1991). Fibronectin: From gene to protein. Curr. Opin. Cell Biol..

[49] Singh P., Carraher C., Schwarzbauer J.E. (2010). Assembly of Fibronectin Extracellular Matrix. Annu. Rev. Cell. Dev. Biol..

[50] Kumra H., Reinhardt D.P. (2016). Fibronectin-targeted drug delivery in cancer. Adv. Drug Deliv. Rev..

[51] Smith H.S., Riggs J.L., Mosesson M.W. (1979). Production of fibronectin by human epithelial cells in culture. Cancer Res..

[52] Vaheri A., Kurkinen M., Lehto V.P., Linder E., Timpl R. (1978). Codistribution of pericellular matrix proteins in cultured fibroblasts and loss in transformation: Fibronectin and procollagen. Proc. Natl. Acad. Sci. USA.

[53] Vaheri A., Mosher D.F. (1978). High molecular weight, cell surface-associated glycoprotein (fibronectin) lost in malignant transformation. Biochim. Biophys. Acta.

[54] Kahn P., Shin S.I. (1979). Cellular tumorigenicity in nude mice. Test of associations among loss of cell-surface fibronectin, anchorage independence, and tumor-forming ability. J. Cell Biol..

[55] Chen L.B., Gallimore P.H., McDougall J.K. (1976). Correlation between tumor induction and the large external transformation sensitive protein on the cell surface. Proc. Natl. Acad. Sci. USA.

[56] Gallimore P.H., McDougall J.K., Chen L.B. (1977). In vitro traits of adenovirus-transformed cell lines and their relevance to tumorigenicity in nude mice. Cell.

[57] Hynes R.O., Destree A.T., Perkins M.E., Wagner D.D. (1979). Cell surface fibronectin and oncogenic transformation. J. Supramol. Struct..

[58] Hayman E.G., Engvall E., Ruoslahti E. (1981). Concomitant loss of cell surface fibronectin and laminin from transformed rat kidney cells. J. Cell Biol..

[59] Hayman E.G., Oldberg A., Martin G.R., Ruoslahti E. (1982). Codistribution of heparan sulfate proteoglycan, laminin, and fibronectin in the extracellular matrix of normal rat kidney cells and their coordinate absence in transformed cells. J. Cell Biol..

[60] Hayman E.G., Engvall E., Ruoslahti E. (1980). Butyrate restores fibronectin at cell surface of transformed cells. Exp. Cell Res..

[61] Akamatsu H., Ichihara-Tanaka K., Ozono K., Kamiike W., Matsuda H., Sekiguchi K. (1996). Suppression of transformed phenotypes of human fibrosarcoma cells by overexpression of recombinant fibronectin. Cancer Res..

[62] Giancotti F.G., Ruoslahti E. (1990). Elevated levels of the alpha 5 beta 1 fibronectin receptor suppress the transformed phenotype of Chinese hamster ovary cells. Cell.

[63] McDoniels-Silvers A.L., Nimri C.F., Stoner G.D., Lubet R.A., You M. (2002). Differential gene expression in human lung adenocarcinomas and squamous cell carcinomas. Clin. Cancer. Res..

[64] Gawecka J.E., Young-Robbins S.S., Sulzmaier F.J., Caliva M.J., Heikkila M.M., Matter M.L., Ramos J.W. (2012). RSK2 protein suppresses integrin activation and fibronectin matrix assembly and promotes cell migration. J. Biol. Chem..

[65] Sabari J., Lax D., Connors D., Brotman I., Mindrebo E., Butler C., Entersz I., Jia D., Foty R.A. (2011). Fibronectin matrix assembly suppresses dispersal of glioblastoma cells. PLoS ONE.

[66] Jia D., Entersz I., Butler C., Foty R.A. (2012). Fibronectin matrix-mediated cohesion suppresses invasion of prostate cancer cells. BMC Cancer.

[67] Colombi M., Zoppi N., De Petro G., Marchina E., Gardella R., Tavian D., Ferraboli S., Barlati S. (2003). Matrix assembly induction and cell migration and invasion inhibition by a 13-amino acid fibronectin peptide. J. Biol. Chem..

[68] Kapoor C., Vaidya S., Wadhwan V., Kaur G., Pathak A. (2016). Seesaw of matrix metalloproteinases (MMPs). J. Cancer Res. Ther..

[69] Wang K., Seo B.R., Fischbach C., Gourdon D. (2016). Fibronectin Mechanobiology Regulates Tumorigenesis. Cell. Mol. Bioeng..

[70] Grither W.R., Divine L.M., Meller E.H., Wilke D.J., Desai R.A., Loza A.J., Zhao P., Lohrey A., Longmore G.D., Fuh K.C. (2018). TWIST1 induces expression of discoidin domain receptor 2 to promote ovarian cancer metastasis. Oncogene.

[71] Ohh M., Yauch R.L., Lonergan K.M., Whaley J.M., Stemmer-Rachamimov A.O., Louis D.N., Gavin B.J., Kley N., Kaelin W.G., Iliopoulos O. (1998). The von Hippel-Lindau tumor suppressor protein is required for proper assembly of an extracellular fibronectin matrix. Mol. Cell.

[72] Lonergan K.M., Iliopoulos O., Ohh M., Kamura T., Conaway R.C., Conaway J.W., Kaelin W.G. (1998). Regulation of hypoxia-inducible mRNAs by the von Hippel-Lindau tumor suppressor protein requires binding to complexes containing elongins B/C and Cul2. Mol. Cell. Biol..

[73] Kamura T., Koepp D.M., Conrad M.N., Skowyra D., Moreland R.J., Iliopoulos O., Lane W.S., Kaelin W.G., Elledge S.J., Conaway R.C. (1999). Rbx1, a component of the VHL tumor suppressor complex and SCF ubiquitin ligase. Science.

[74] Pause A., Lee S., Worrell R.A., Chen D.Y., Burgess W.H., Linehan W.M., Klausner R.D. (1997). The von Hippel-Lindau tumor-suppressor gene product forms a stable complex with human CUL-2, a member of the Cdc53 family of proteins. Proc. Natl. Acad. Sci. USA.

[75] Cheng H.C., Abdel-Ghany M., Elble R.C., Pauli B.U. (1998). Lung endothelial dipeptidyl peptidase IV promotes adhesion and metastasis of rat breast cancer cells via tumor cell surface-associated fibronectin. J. Biol. Chem..

[76] Cheng H.C., Abdel-Ghany M., Zhang S., Pauli B.U. (1999). Is the Fischer 344/CRJ rat a protein-knock-out model for dipeptidyl peptidase IV-mediated lung metastasis of breast cancer?. Clin. Exp. Metastasis.

[77] Clark E.A., Golub T.R., Lander E.S., Hynes R.O. (2000). Genomic analysis of metastasis reveals an essential role for RhoC. Nature.

[78] Han H.-J., Russo J., Kohwi Y., Kohwi-Shigematsu T. (2008). SATB1 reprogrammes gene expression to promote breast tumour growth and metastasis. Nature.

[79] Wong F.H., Huang C.Y., Su L.J., Wu Y.C., Lin Y.S., Hsia J.Y., Tsai H.T., Lee S.A., Lin C.H., Tzeng C.H. (2009). Combination of microarray profiling and protein-protein interaction databases delineates the minimal discriminators as a metastasis network for esophageal squamous cell carcinoma. Int. J. Oncol..

[80] Reuter J.A., Ortiz-Urda S., Kretz M., Garcia J., Scholl F.A., Pasmooij A.M., Cassarino D., Chang H.Y., Khavari P.A. (2009). Modeling inducible human tissue neoplasia identifies an extracellular matrix interaction network involved in cancer progression. Cancer Cell.

[81] Steffens S., Schrader A.J., Vetter G., Eggers H., Blasig H., Becker J., Kuczyk M.A., Serth J. (2012). Fibronectin 1 protein expression in clear cell renal cell carcinoma. Oncol. Lett..

[82] Ma L.J., Lee S.W., Lin L.C., Chen T.J., Chang I.W., Hsu H.P., Chang K.Y., Huang H.Y., Li C.F. (2014). Fibronectin overexpression is associated with latent membrane protein 1 expression and has independent prognostic value for nasopharyngeal carcinoma. Tumor Biol..

[83] Li Y., Miao L., Yu M., Shi M., Wang Y., Yang J., Xiao Y., Cai H. (2017). alpha1-antitrypsin promotes lung adenocarcinoma metastasis through upregulating fibronectin expression. Int. J. Oncol..

[84] Fernandez-Garcia B., Eiro N., Marin L., Gonzalez-Reyes S., Gonzalez L.O., Lamelas M.L., Vizoso F.J. (2014). Expression and prognostic significance of fibronectin and matrix metalloproteases in breast cancer metastasis. Histopathology.

[85] Kenny H.A., Chiang C.Y., White E.A., Schryver E.M., Habis M., Romero I.L., Ladanyi A., Penicka C.V., George J., Matlin K. (2014). Mesothelial cells promote early ovarian cancer metastasis through fibronectin secretion. J. Clin. Investig..

[86] Niknami Z., Eslamifar A., Emamirazavi A., Ebrahimi A., Shirkoohi R. (2017). The association of vimentin and fibronectin gene expression with epithelial-mesenchymal transition and tumor malignancy in colorectal carcinoma. Excli. J..

[87] Hegele A., Heidenreich A., Kropf J., von Knobloch R., Varga Z., Hofmann R., Olbert P. (2004). Plasma levels of cellular fibronectin in patients with localized and metastatic renal cell carcinoma. Tumor Biol..

[88] Saito N., Nishimura H., Kameoka S. (2008). Clinical significance of fibronectin expression in colorectal cancer. Mol. Med. Rep..

[89] Yu M., Ting D.T., Stott S.L., Wittner B.S., Ozsolak F., Paul S., Ciciliano J.C., Smas M.E., Winokur D., Gilman A.J. (2012). RNA sequencing of pancreatic circulating tumor cells implicates WNT signalling in metastasis. Nature.

[90] Xu T.P., Huang M.D., Xia R., Liu X.X., Sun M., Yin L., Chen W.M., Han L., Zhang E.B., Kong R. (2014). Decreased expression of the long non-coding RNA FENDRR is associated with poor prognosis in gastric cancer and FENDRR regulates gastric cancer cell metastasis by affecting fibronectin1 expression. J. Hematol. Oncol..

[91] Lee H.A., Park I., Byun H.J., Jeoung D., Kim Y.M., Lee H. (2011). Metastasis suppressor KAI1/CD82 attenuates the matrix adhesion of human prostate cancer cells by suppressing fibronectin expression and beta1 integrin activation. Cell. Physiol. Biochem..

[92] Mehrotra S., Languino L.R., Raskett C.M., Mercurio A.M., Dohi T., Altieri D.C. (2010). IAP regulation of metastasis. Cancer Cell.

[93] Meier C., Spitschak A., Abshagen K., Gupta S., Mor J.M., Wolkenhauer O., Haier J., Vollmar B., Alla V., Putzer B.M. (2014). Association of RHAMM with E2F1 promotes tumor cell extravasation by transcriptional up-regulation of fibronectin. J. Pathol..

[94] Hong H., Zhou T., Fang S., Jia M., Xu Z., Dai Z., Li C., Li S., Li L., Zhang T. (2014). Pigment epithelium-derived factor (PEDF) inhibits breast cancer metastasis by down-regulating fibronectin. Breast Cancer Res. Treat..

[95] Rhim A.D., Mirek E.T., Aiello N.M., Maitra A., Bailey J.M., McAllister F., Reichert M., Beatty G.L., Rustgi A.K., Vonderheide R.H. (2012). EMT and dissemination precede pancreatic tumor formation. Cell.

[96] Zhang Y., Wang X.F. (2015). A niche role for cancer exosomes in metastasis. Nat. Cell Biol..

[97] Costa-Silva B., Aiello N.M., Ocean A.J., Singh S., Zhang H., Thakur B.K., Becker A., Hoshino A., Mark M.T., Molina H. (2015). Pancreatic cancer exosomes initiate pre-metastatic niche formation in the liver. Nat. Cell Biol..

[98] Lan T., Chang L., Wu L., Yuan Y. (2016). Downregulation of ZEB2-AS1 decreased tumor growth and metastasis in hepatocellular carcinoma. Mol. Med. Rep..

[99] Hsu J.Y., Chang J.Y., Chang K.Y., Chang W.C., Chen B.K. (2017). Epidermal growth factor-induced pyruvate dehydrogenase kinase 1 expression enhances head and neck squamous cell carcinoma metastasis via up-regulation of fibronectin. FASEB J..

[100] Di Modugno F., Spada S., Palermo B., Visca P., Iapicca P., Di Carlo A., Antoniani B., Sperduti I., Di Benedetto A., Terrenato I. (2018). hMENA isoforms impact NSCLC patient outcome through fibronectin/beta1 integrin axis. Oncogene.

[101] Morita Y., Hata K., Nakanishi M., Omata T., Morita N., Yura Y., Nishimura R., Yoneda T. (2015). Cellular fibronectin 1 promotes VEGF-C expression, lymphangiogenesis and lymph node metastasis associated with human oral squamous cell carcinoma. Clin. Exp. Metastasis.

[102] Jagadeeshan S., Krishnamoorthy Y.R., Singhal M., Subramanian A., Mavuluri J., Lakshmi A., Roshini A., Baskar G., Ravi M., Joseph L.D. (2015). Transcriptional regulation of fibronectin by p21-activated kinase-1 modulates pancreatic tumorigenesis. Oncogene.

[103] Sengupta S., Nandi S., Hindi E.S., Wainwright D.A., Han Y., Lesniak M.S. (2010). Short hairpin RNA-mediated fibronectin knockdown delays tumor growth in a mouse glioma model. Neoplasia.

[104] Liao Y.X., Zhang Z.P., Zhao J., Liu J.P. (2018). Effects of Fibronectin 1 on Cell Proliferation, Senescence and Apoptosis of Human Glioma Cells Through the PI3K/AKT Signaling Pathway. Cell. Physiol. Biochem..

[105] Shinde A., Libring S., Alpsoy A., Abdullah A., Schaber J.A., Solorio L., Wendt M.K. (2018). Autocrine Fibronectin Inhibits Breast Cancer Metastasis. Mol. Cancer Res..

[106] Liu W., Cheng S., Asa S.L., Ezzat S. (2008). The melanoma-associated antigen A3 mediates fibronectin-controlled cancer progression and metastasis. Cancer Res..

[107] Calcinotto A., Kohli J., Zagato E., Pellegrini L., Demaria M., Alimonti A. (2019). Cellular Senescence: Aging, Cancer, and Injury. Physiol. Rev..

[108] He S., Sharpless N.E. (2017). Senescence in Health and Disease. Cell.

[109] Hernandez-Segura A., Nehme J., Demaria M. (2018). Hallmarks of Cellular Senescence. Trends Cell Biol..

[110] Mazzucco A.E., Smogorzewska A., Kang C., Luo J., Schlabach M.R., Xu Q., Patel R., Elledge S.J. (2017). Genetic interrogation of replicative senescence uncovers a dual role for USP28 in coordinating the p53 and GATA4 branches of the senescence program. Genes Dev..

[111] Acosta J.C., Gil J. (2012). Senescence: A new weapon for cancer therapy. Trends Cell Biol..

[112] Canino C., Mori F., Cambria A., Diamantini A., Germoni S., Alessandrini G., Borsellino G., Galati R., Battistini L., Blandino R. (2012). SASP mediates chemoresistance and tumor-initiating-activity of mesothelioma cells. Oncogene.

[113] Coppe J.P., Desprez P.Y., Krtolica A., Campisi J. (2010). The senescence-associated secretory phenotype: The dark side of tumor suppression. Annu. Rev. Pathol..

[114] Hoare M., Narita M. (2018). The Power Behind the Throne: Senescence and the Hallmarks of Cancer. Annu. Rev. Cancer Biol..

[115] Ksiazek K., Mikula-Pietrasik J., Korybalska K., Dworacki G., Jorres A., Witowski J. (2009). Senescent peritoneal mesothelial cells promote ovarian cancer cell adhesion: The role of oxidative stress-induced fibronectin. Am. J. Pathol..

[116] Ruggiano A., Foresti O., Carvalho P. (2014). Quality control: ER-associated degradation: Protein quality control and beyond. J. Cell Biol..

[117] Kaufman R.J. (1999). Stress signaling from the lumen of the endoplasmic reticulum: Coordination of gene transcriptional and translational controls. Genes Dev..

[118] Pluquet O., Pourtier A., Abbadie C. (2015). The unfolded protein response and cellular senescence. A review in the theme: Cellular mechanisms of endoplasmic reticulum stress signaling in health and disease. Am. J. Physiol. Cell Physiol..

[119] Courtois-Cox S., Genther Williams S.M., Reczek E.E., Johnson B.W., McGillicuddy L.T., Johannessen C.M., Hollstein P.E., MacCollin M., Cichowski K. (2006). A negative feedback signaling network underlies oncogene-induced senescence. Cancer Cell.

[120] Wu C.H., van Riggelen J., Yetil A., Fan A.C., Bachireddy P., Felsher D.W. (2007). Cellular senescence is an important mechanism of tumor regression upon c-Myc inactivation. Proc. Natl. Acad. Sci. USA.

[121] Mooi W.J., Peeper D.S. (2006). Oncogene-induced cell senescence--halting on the road to cancer. N. Engl. J. Med..

[122] Collado M., Serrano M. (2010). Senescence in tumours: Evidence from mice and humans. Nat. Rev. Cancer.

[123] Kuilman T., Michaloglou C., Mooi W.J., Peeper D.S. (2010). The essence of senescence. Genes Dev..

[124] Glasner A., Levi A., Enk J., Isaacson B., Viukov S., Orlanski S., Scope A., Neuman T., Enk C.D., Hanna J.H. (2018). NKp46 Receptor-Mediated Interferon-gamma Production by Natural Killer Cells Increases Fibronectin 1 to Alter Tumor Architecture and Control Metastasis. Immunity.

[125] Novak M., Leonard M.K., Yang X.H., Kowluru A., Belkin A.M., Kaetzel D.M. (2015). Metastasis suppressor NME1 regulates melanoma cell morphology, self-adhesion and motility via induction of fibronectin expression. Exp. Dermatol..

[126] Brentnall M., Weir D.B., Rongvaux A., Marcus A.I., Boise L.H. (2014). Procaspase-3 regulates fibronectin secretion and influences adhesion, migration and survival independently of catalytic function. J. Cell Sci..

[127] Korybalska K., Kawka E., Kusch A., Aregger F., Dragun D., Jorres A., Breborowicz A., Witowski J. (2013). Recovery of senescent endothelial cells from injury. J. Gerontol. A Biol. Sci. Med. Sci..

[128] Gorospe M., Egan J.M., Zbar B., Lerman M., Geil L., Kuzmin I., Holbrook N.J. (1999). Protective function of von Hippel-Lindau protein against impaired protein processing in renal carcinoma cells. Mol. Cell. Biol..

[129] Pause A., Peterson B., Schaffar G., Stearman R., Klausner R.D. (1999). Studying interactions of four proteins in the yeast two-hybrid system: Structural resemblance of the pVHL/elongin BC/hCUL-2 complex with the ubiquitin ligase complex SKP1/cullin/F-box protein. Proc. Natl. Acad. Sci. USA.

[130] Kaelin W.G., Maher E.R. (1998). The VHL tumour-suppressor gene paradigm. Trends Genet..

[131] Guilford P. (2000). The inherited susceptibility to cancer. Cell. Mol. Life Sci..

[132] Armstrong H.K., Gillis J.L., Johnson I.R.D., Nassar Z.D., Moldovan M., Levrier C., Sadowski M.C., Chin M.Y., Tomlinson Guns E.S., Tarulli G. (2018). Dysregulated fibronectin trafficking by Hsp90 inhibition restricts prostate cancer cell invasion. Sci. Rep..

[133] Hunter M.C., O’Hagan K.L., Kenyon A., Dhanani K.C., Prinsloo E., Edkins A.L. (2014). Hsp90 binds directly to fibronectin (FN) and inhibition reduces the extracellular fibronectin matrix in breast cancer cells. PLoS ONE.

[134] Han S.Y., Ko A., Kitano H., Choi C.H., Lee M.S., Seo J., Fukuoka J., Kim S.Y., Hewitt S.M., Chung J.Y. (2017). Molecular Chaperone HSP90 Is Necessary to Prevent Cellular Senescence via Lysosomal Degradation of p14ARF. Cancer Res..

[135] Schosserer M., Grillari J., Breitenbach M. (2017). The Dual Role of Cellular Senescence in Developing Tumors and Their Response to Cancer Therapy. Front. Oncol..

[136] Eggert T., Wolter K., Ji J., Ma C., Yevsa T., Klotz S., Medina-Echeverz J., Longerich T., Forgues M., Reisinger F. (2016). Distinct Functions of Senescence-Associated Immune Responses in Liver Tumor Surveillance and Tumor Progression. Cancer Cell.

[137] Poggi A., Musso A., Dapino I., Zocchi M.R. (2014). Mechanisms of tumor escape from immune system: Role of mesenchymal stromal cells. Immunol. Lett..

[138] Chang C.H., Qiu J., O’Sullivan D., Buck M.D., Noguchi T., Curtis J.D., Chen Q., Gindin M., Gubin M.M., van der Windt G.J. (2015). Metabolic Competition in the Tumor Microenvironment Is a Driver of Cancer Progression. Cell.

[139] Liotta L.A., Rao C.N., Wewer U.M. (1986). Biochemical interactions of tumor cells with the basement membrane. Annu. Rev. Biochem..

[140] Liu Z.C., Ning F., Wang H.F., Chen D.Y., Cai Y.N., Sheng H.Y., Lash G.E., Liu L., Du J. (2017). Epidermal growth factor and tumor necrosis factor alpha cooperatively promote the motility of hepatocellular carcinoma cell lines via synergistic induction of fibronectin by NF-kappaB/p65. Biochim. Biophys. Acta Gen. Subj..

[141] Cao Y., Liu X., Lu W., Chen Y., Wu X., Li M., Wang X.A., Zhang F., Jiang L., Zhang Y. (2015). Fibronectin promotes cell proliferation and invasion through mTOR signaling pathway activation in gallbladder cancer. Cancer Lett..

[142] Yousif N.G. (2014). Fibronectin promotes migration and invasion of ovarian cancer cells through up-regulation of FAK-PI3K/Akt pathway. Cell Biol. Int..

[143] Pereira I.T., Ramos E.A., Costa E.T., Camargo A.A., Manica G.C., Klassen L.M., Chequin A., Braun-Prado K., Pedrosa Fde O., Souza E.M. (2014). Fibronectin affects transient MMP2 gene expression through DNA demethylation changes in non-invasive breast cancer cell lines. PLoS ONE.

[144] Schafer M.J., White T.A., Iijima K., Haak A.J., Ligresti G., Atkinson E.J., Oberg A.L., Birch J., Salmonowicz H., Zhu Y. (2017). Cellular senescence mediates fibrotic pulmonary disease. Nat. Commun..

[145] Yamauchi M., Barker T.H., Gibbons D.L., Kurie J.M. (2018). The fibrotic tumor stroma. J. Clin. Investig..

[146] Kramer R.H., Gonzalez R., Nicolson G.L. (1980). Metastatic tumor cells adhere preferentially to the extracellular matrix underlying vascular endothelial cells. Int. J. Cancer.

[147] Naba A., Clauser K.R., Mani D.R., Carr S.A., Hynes R.O. (2017). Quantitative proteomic profiling of the extracellular matrix of pancreatic islets during the angiogenic switch and insulinoma progression. Sci. Rep..

[148] Kai F., Drain A.P., Weaver V.M. (2019). The Extracellular Matrix Modulates the Metastatic Journey. Dev. Cell.

[149] Gauperaa T., Seljelid R. (1986). Plasma fibronectin is sequestered into tissue damaged by inflammation and trauma. Acta Chir. Scand..

[150] Fang T., Lv H., Lv G., Li T., Wang C., Han Q., Yu L., Su B., Guo L., Huang S. (2018). Tumor-derived exosomal miR-1247-3p induces cancer-associated fibroblast activation to foster lung metastasis of liver cancer. Nat. Commun..

[151] Trani M., Dejana E. (2015). New insights in the control of vascular permeability: Vascular endothelial-cadherin and other players. Curr. Opin. Hematol..

[152] Goel S., Duda D.G., Xu L., Munn L.L., Boucher Y., Fukumura D., Jain R.K. (2011). Normalization of the vasculature for treatment of cancer and other diseases. Physiol. Rev..

[153] Schafer M., Werner S. (2008). Cancer as an overhealing wound: An old hypothesis revisited. Nat. Rev. Mol. Cell Biol..

[154] Weis S.M. (2008). Vascular permeability in cardiovascular disease and cancer. Curr. Opin. Hematol..

[155] Dvorak H.F. (2010). Vascular permeability to plasma, plasma proteins, and cells: An update. Curr. Opin. Hematol..

[156] De Palma M., Biziato D., Petrova T.V. (2017). Microenvironmental regulation of tumour angiogenesis. Nat. Rev. Cancer.

[157] Carmeliet P., Jain R.K. (2000). Angiogenesis in cancer and other diseases. Nature.

[158] Wang Y., Reheman A., Spring C.M., Kalantari J., Marshall A.H., Wolberg A.S., Gross P.L., Weitz J.I., Rand M.L., Mosher D.F. (2014). Plasma fibronectin supports hemostasis and regulates thrombosis. J. Clin. Investig..

[159] Tao L., Huang G., Song H., Chen Y., Chen L. (2017). Cancer associated fibroblasts: An essential role in the tumor microenvironment. Oncol. Lett..

[160] Schafer M.J., Haak A.J., Tschumperlin D.J., LeBrasseur N.K. (2018). Targeting Senescent Cells in Fibrosis: Pathology, Paradox, and Practical Considerations. Curr. Rheumatol. Rep..

[161] Takaoka A., Taniguchi T. (2003). New aspects of IFN-alpha/beta signalling in immunity, oncogenesis and bone metabolism. Cancer Sci..

[162] Coppe J.P., Patil C.K., Rodier F., Sun Y., Munoz D.P., Goldstein J., Nelson P.S., Desprez P.Y., Campisi J. (2008). Senescence-associated secretory phenotypes reveal cell-nonautonomous functions of oncogenic RAS and the p53 tumor suppressor. PloS Biol..

[163] Moiseeva O., Bourdeau V., Roux A., Deschenes-Simard X., Ferbeyre G. (2009). Mitochondrial dysfunction contributes to oncogene-induced senescence. Mol. Cell. Biol..

[164] Mavrogonatou E., Pratsinis H., Papadopoulou A., Karamanos N.K., Kletsas D. (2019). Extracellular matrix alterations in senescent cells and their significance in tissue homeostasis. Matrix Biol..

[165] Altrock E., Sens C., Wuerfel C., Vasel M., Kawelke N., Dooley S., Sottile J., Nakchbandi I.A. (2015). Inhibition of fibronectin deposition improves experimental liver fibrosis. J. Hepatol..

[166] Saneyasu T., Akhtar R., Sakai T. (2016). Molecular Cues Guiding Matrix Stiffness in Liver Fibrosis. Biomed Res. Int..

[167] Yang Z., Xiaohua W., Lei J., Ruoyun T., Mingxia X., Weichun H., Li F., Ping W., Junwei Y. (2010). Uric acid increases fibronectin synthesis through upregulation of lysyl oxidase expression in rat renal tubular epithelial cells. Am. J. Physiol. Ren. Physiol..

[168] Funk W.D., Wang C.K., Shelton D.N., Harley C.B., Pagon G.D., Hoeffler W.K. (2000). Telomerase expression restores dermal integrity to in vitro-aged fibroblasts in a reconstituted skin model. Exp. Cell Res..

[169] Pratsinis H., Armatas A., Dimozi A., Lefaki M., Vassiliu P., Kletsas D. (2013). Paracrine anti-fibrotic effects of neonatal cells and living cell constructs on young and senescent human dermal fibroblasts. Wound Repair Regen..

[170] Kamino H., Hiratsuka M., Toda T., Nishigaki R., Osaki M., Ito H., Inoue T., Oshimura M. (2003). Searching for genes involved in arteriosclerosis: Proteomic analysis of cultured human umbilical vein endothelial cells undergoing replicative senescence. Cell Struct. Funct..

[171] Yin H., Pickering J.G. (2016). Cellular Senescence and Vascular Disease: Novel Routes to Better Understanding and Therapy. Can. J. Cardiol..

[172] Solinas G., Schiarea S., Liguori M., Fabbri M., Pesce S., Zammataro L., Pasqualini F., Nebuloni M., Chiabrando C., Mantovani A. (2010). Tumor-conditioned macrophages secrete migration-stimulating factor: A new marker for M2-polarization, influencing tumor cell motility. J. Immunol..

[173] Alitalo K., Hovi T., Vaheri A. (1980). Fibronectin is produced by human macrophages. J. Exp. Med..

[174] Schmieder A., Schledzewski K., Michel J., Tuckermann J.P., Tome L., Sticht C., Gkaniatsou C., Nicolay J.P., Demory A., Faulhaber J. (2011). Synergistic activation by p38MAPK and glucocorticoid signaling mediates induction of M2-like tumor-associated macrophages expressing the novel CD20 homolog MS4A8A. Int. J. Cancer.

[175] Martinez F.O., Gordon S., Locati M., Mantovani A. (2006). Transcriptional profiling of the human monocyte-to-macrophage differentiation and polarization: New molecules and patterns of gene expression. J. Immunol..

[176] Li Q., Zheng M., Liu Y., Sun W., Shi J., Ni J., Wang Q. (2018). A pathogenetic role for M1 macrophages in peritoneal dialysis-associated fibrosis. Mol. Immunol..

[177] Gratchev A., Guillot P., Hakiy N., Politz O., Orfanos C.E., Schledzewski K., Goerdt S. (2001). Alternatively activated macrophages differentially express fibronectin and its splice variants and the extracellular matrix protein betaIG-H3. Scand. J. Immunol..

[178] Fiori M.E., Villanova L., De Maria R. (2017). Cancer stem cells: At the forefront of personalized medicine and immunotherapy. Curr. Opin. Pharmacol..

[179] Qian B.Z., Pollard J.W. (2010). Macrophage diversity enhances tumor progression and metastasis. Cell.

[180] Quail D.F., Joyce J.A. (2013). Microenvironmental regulation of tumor progression and metastasis. Nat. Med..

[181] Sangaletti S., Di Carlo E., Gariboldi S., Miotti S., Cappetti B., Parenza M., Rumio C., Brekken R.A., Chiodoni C., Colombo M.P. (2008). Macrophage-derived SPARC bridges tumor cell-extracellular matrix interactions toward metastasis. Cancer Res..

[182] Bradshaw A.D. (2016). The role of secreted protein acidic and rich in cysteine (SPARC) in cardiac repair and fibrosis: Does expression of SPARC by macrophages influence outcomes?. J. Mol. Cell. Cardiol..

[183] Ojalvo L.S., King W., Cox D., Pollard J.W. (2009). High-density gene expression analysis of tumor-associated macrophages from mouse mammary tumors. Am. J. Pathol..

[184] Zabuawala T., Taffany D.A., Sharma S.M., Merchant A., Adair B., Srinivasan R., Rosol T.J., Fernandez S., Huang K., Leone G. (2010). An ets2-driven transcriptional program in tumor-associated macrophages promotes tumor metastasis. Cancer Res..

[185] Pollard J.W. (2009). Trophic macrophages in development and disease. Nat. Rev. Immunol..

[186] Mantovani A., Sica A. (2010). Macrophages, innate immunity and cancer: Balance, tolerance, and diversity. Curr. Opin. Immunol..

[187] Omigbodun A., Coukos G., Ziolkiewicz P., Wang C.L., Coutifaris C. (1998). Macrophage-colony stimulating factor (M-CSF) regulates the expression of fibronectin and its alpha5 integrin receptor in human trophoblasts. Endocrinology.

[188] Huang L., Xu X., Hao Y. (2014). The possible mechanisms of tumor progression via CSF-1/CSF-1R pathway activation. Rom. J. Morphol. Embryol..

[189] Yoshidome H., Kohno H., Shida T., Kimura F., Shimizu H., Ohtsuka M., Nakatani Y., Miyazaki M. (2009). Significance of monocyte chemoattractant protein-1 in angiogenesis and survival in colorectal liver metastases. Int. J. Oncol..

[190] Zijlmans H.J., Fleuren G.J., Baelde H.J., Eilers P.H., Kenter G.G., Gorter A. (2006). The absence of CCL2 expression in cervical carcinoma is associated with increased survival and loss of heterozygosity at 17q11.2. J. Pathol..

[191] Chen W., Gao Q., Han S., Pan F., Fan W. (2015). The CCL2/CCR2 axis enhances IL-6-induced epithelial-mesenchymal transition by cooperatively activating STAT3-Twist signaling. Tumor Biol..

[192] Ray S., Ju X., Sun H., Finnerty C.C., Herndon D.N., Brasier A.R. (2013). The IL-6 trans-signaling-STAT3 pathway mediates ECM and cellular proliferation in fibroblasts from hypertrophic scar. J. Invest. Dermatol..

[193] Zhang F., Li C., Halfter H., Liu J. (2003). Delineating an oncostatin M-activated STAT3 signaling pathway that coordinates the expression of genes involved in cell cycle regulation and extracellular matrix deposition of MCF-7 cells. Oncogene.

[194] Biswas S.K., Mantovani A. (2010). Macrophage plasticity and interaction with lymphocyte subsets: Cancer as a paradigm. Nat. Immunol..

[195] Mantovani A., Biswas S.K., Galdiero M.R., Sica A., Locati M. (2013). Macrophage plasticity and polarization in tissue repair and remodelling. J. Pathol..

[196] Mosser D.M., Edwards J.P. (2008). Exploring the full spectrum of macrophage activation. Nat. Rev. Immunol..

[197] Hao N.-B., Lü M.-H., Fan Y.-H., Cao Y.-L., Zhang Z.-R., Yang S.-M. (2012). Macrophages in Tumor Microenvironments and the Progression of Tumors. Clin. Dev. Immunol..

[198] Franklin R.A., Liao W., Sarkar A., Kim M.V., Bivona M.R., Liu K., Pamer E.G., Li M.O. (2014). The cellular and molecular origin of tumor-associated macrophages. Science.

[199] Gilkes D.M., Semenza G.L., Wirtz D. (2014). Hypoxia and the extracellular matrix: Drivers of tumour metastasis. Nat. Rev. Cancer.

[200] Vaupel P., Mayer A. (2014). Hypoxia in tumors: Pathogenesis-related classification, characterization of hypoxia subtypes, and associated biological and clinical implications. Adv. Exp. Med. Biol..

[201] Semenza G.L. (2012). Molecular mechanisms mediating metastasis of hypoxic breast cancer cells. Trends Mol. Med..

[202] Luoto K.R., Kumareswaran R., Bristow R.G. (2013). Tumor hypoxia as a driving force in genetic instability. Genome Integr..

[203] Xie H., Simon M.C. (2017). Oxygen availability and metabolic reprogramming in cancer. J. Biol. Chem..

[204] Nakazawa M.S., Keith B., Simon M.C. (2016). Oxygen availability and metabolic adaptations. Nat. Rev. Cancer.

[205] Balamurugan K. (2016). HIF-1 at the crossroads of hypoxia, inflammation, and cancer. Int. J. Cancer.

[206] Joseph J.V., Conroy S., Pavlov K., Sontakke P., Tomar T., Eggens-Meijer E., Balasubramaniyan V., Wagemakers M., den Dunnen W.F., Kruyt F.A. (2015). Hypoxia enhances migration and invasion in glioblastoma by promoting a mesenchymal shift mediated by the HIF1alpha-ZEB1 axis. Cancer Lett..

[207] Nurwidya F., Takahashi F., Kobayashi I., Murakami A., Kato M., Minakata K., Nara T., Hashimoto M., Yagishita S., Baskoro H. (2014). Treatment with insulin-like growth factor 1 receptor inhibitor reverses hypoxia-induced epithelial-mesenchymal transition in non-small cell lung cancer. Biochem. Biophys. Res. Commun..

[208] Lee S.H., Lee Y.J., Han H.J. (2011). Role of hypoxia-induced fibronectin-integrin beta1 expression in embryonic stem cell proliferation and migration: Involvement of PI3K/Akt and FAK. J. Cell. Physiol..

[209] Ryu M.H., Park H.M., Chung J., Lee C.H., Park H.R. (2010). Hypoxia-inducible factor-1alpha mediates oral squamous cell carcinoma invasion via upregulation of alpha5 integrin and fibronectin. Biochem. Biophys. Res. Commun..

[210] Gossage L., Eisen T., Maher E.R. (2015). VHL, the story of a tumour suppressor gene. Nat. Rev. Cancer.

[211] Crespigio J., Berbel L.C.L., Dias M.A., Berbel R.F., Pereira S.S., Pignatelli D., Mazzuco T.L. (2018). Von Hippel-Lindau disease: A single gene, several hereditary tumors. J. Endocrinol. Investig..

[212] Stickle N.H., Chung J., Klco J.M., Hill R.P., Kaelin W.G., Ohh M. (2004). pVHL modification by NEDD8 is required for fibronectin matrix assembly and suppression of tumor development. Mol. Cell. Biol..

[213] Distler J.H., Jungel A., Pileckyte M., Zwerina J., Michel B.A., Gay R.E., Kowal-Bielecka O., Matucci-Cerinic M., Schett G., Marti H.H. (2007). Hypoxia-induced increase in the production of extracellular matrix proteins in systemic sclerosis. Arthritis Rheum..

[214] Yeung K.T., Yang J. (2017). Epithelial-mesenchymal transition in tumor metastasis. Mol. Oncol..

[215] Suarez-Carmona M., Lesage J., Cataldo D., Gilles C. (2017). EMT and inflammation: Inseparable actors of cancer progression. Mol. Oncol..

[216] Shibue T., Weinberg R.A. (2017). EMT, CSCs, and drug resistance: The mechanistic link and clinical implications. Nat. Rev. Clin. Oncol..

[217] Celia-Terrassa T., Kang Y. (2016). Distinctive properties of metastasis-initiating cells. Genes Dev..

[218] Weidenfeld K., Barkan D. (2018). EMT and Stemness in Tumor Dormancy and Outgrowth: Are They Intertwined Processes?. Front. Oncol..

[219] Li Y., Rogoff H.A., Keates S., Gao Y., Murikipudi S., Mikule K., Leggett D., Li W., Pardee A.B., Li C.J. (2015). Suppression of cancer relapse and metastasis by inhibiting cancer stemness. Proc. Natl. Acad. Sci. USA.

[220] Saygin C., Matei D., Majeti R., Reizes O., Lathia J.D. (2019). Targeting Cancer Stemness in the Clinic: From Hype to Hope. Cell Stem Cell.

[221] Sponziello M., Rosignolo F., Celano M., Maggisano V., Pecce V., De Rose R.F., Lombardo G.E., Durante C., Filetti S., Damante G. (2016). Fibronectin-1 expression is increased in aggressive thyroid cancer and favors the migration and invasion of cancer cells. Mol. Cell. Endocrinol..

[222] Vu T., Datta P.K. (2017). Regulation of EMT in Colorectal Cancer: A Culprit in Metastasis. Cancers.

[223] Ye L.Y., Chen W., Bai X.L., Xu X.Y., Zhang Q., Xia X.F., Sun X., Li G.G., Hu Q.D., Fu Q.H. (2016). Hypoxia-Induced Epithelial-to-Mesenchymal Transition in Hepatocellular Carcinoma Induces an Immunosuppressive Tumor Microenvironment to Promote Metastasis. Cancer Res..

[224] Dimri G.P., Lee X., Basile G., Acosta M., Scott G., Roskelley C., Medrano E.E., Linskens M., Rubelj I., Pereira-Smith O. (1995). A biomarker that identifies senescent human cells in culture and in aging skin in vivo. Proc. Natl. Acad. Sci. USA.

[225] Chen A., Sceneay J., Godde N., Kinwel T., Ham S., Thompson E.W., Humbert P.O., Moller A. (2018). Intermittent hypoxia induces a metastatic phenotype in breast cancer. Oncogene.

[226] You J., Dong R., Ying M., He Q., Cao J., Yang B. (2019). Cellular Senescence and Anti-Cancer Therapy. Curr. Drug Targets.

[227] Gilkes D.M., Xiang L., Lee S.J., Chaturvedi P., Hubbi M.E., Wirtz D., Semenza G.L. (2014). Hypoxia-inducible factors mediate coordinated RhoA-ROCK1 expression and signaling in breast cancer cells. Proc. Natl. Acad. Sci. USA.

[228] Li M., Ye L., Ye X., Wang S., Zhang H., Liu J., Hong H. (2019). Hypoxia-induced ARHGAP26 deficiency inhibits the proliferation and migration of human ductus arteriosus smooth muscle cell through activating RhoA-ROCK-PTEN pathway. J. Cell. Biochem..

[229] Wu W.S., Heinrichs S., Xu D., Garrison S.P., Zambetti G.P., Adams J.M., Look A.T. (2005). Slug antagonizes p53-mediated apoptosis of hematopoietic progenitors by repressing puma. Cell.

[230] Zhang Z., Lee J.C., Lin L., Olivas V., Au V., LaFramboise T., Abdel-Rahman M., Wang X., Levine A.D., Rho J.K. (2012). Activation of the AXL kinase causes resistance to EGFR-targeted therapy in lung cancer. Nat. Genet..

[231] Kudo-Saito C., Shirako H., Takeuchi T., Kawakami Y. (2009). Cancer metastasis is accelerated through immunosuppression during Snail-induced EMT of cancer cells. Cancer Cell.

[232] Chen L., Gibbons D.L., Goswami S., Cortez M.A., Ahn Y.H., Byers L.A., Zhang X., Yi X., Dwyer D., Lin W. (2014). Metastasis is regulated via microRNA-200/ZEB1 axis control of tumour cell PD-L1 expression and intratumoral immunosuppression. Nat. Commun..

[233] Marienfeld C., Yamagiwa Y., Ueno Y., Chiasson V., Brooks L., Meng F., Patel T. (2004). Translational regulation of XIAP expression and cell survival during hypoxia in human cholangiocarcinoma. Gastroenterology.

[234] Wu M., Yuan S., Szporn A.H., Gan L., Shtilbans V., Burstein D.E. (2005). Immunocytochemical detection of XIAP in body cavity effusions and washes. Mod. Pathol..

[235] Ahmed F., Haass N.K. (2018). Microenvironment-Driven Dynamic Heterogeneity and Phenotypic Plasticity as a Mechanism of Melanoma Therapy Resistance. Front. Oncol..

[236] Prieto-Vila M., Takahashi R.U., Usuba W., Kohama I., Ochiya T. (2017). Drug Resistance Driven by Cancer Stem Cells and Their Niche. Int. J. Mol. Sci..

[237] Casazza A., Di Conza G., Wenes M., Finisguerra V., Deschoemaeker S., Mazzone M. (2014). Tumor stroma: A complexity dictated by the hypoxic tumor microenvironment. Oncogene.

[238] Kim I.K., Kim K., Lee E., Oh D.S., Park C.S., Park S., Yang J.M., Kim J.H., Kim H.S., Shima D.T. (2018). Sox7 promotes high-grade glioma by increasing VEGFR2-mediated vascular abnormality. J. Exp. Med..

[239] Chappell J.C., Payne L.B., Rathmell W.K. (2019). Hypoxia, angiogenesis, and metabolism in the hereditary kidney cancers. J. Clin. Investig..

[240] Krock B.L., Skuli N., Simon M.C. (2011). Hypoxia-induced angiogenesis: Good and evil. Genes Cancer.

[241] Murphy P.A., Begum S., Hynes R.O. (2015). Tumor angiogenesis in the absence of fibronectin or its cognate integrin receptors. PLoS ONE.

[242] Kaushik S., Pickup M.W., Weaver V.M. (2016). From transformation to metastasis: Deconstructing the extracellular matrix in breast cancer. Cancer Metastasis Rev..

[243] Han Y.L., Chen L., Qin R., Wang G.Q., Lin X.H., Dai G.H. (2019). Lysyl oxidase and hypoxia-inducible factor 1alpha: Biomarkers of gastric cancer. World J. Gastroenterol..

[244] Rossow L., Veitl S., Vorlova S., Wax J.K., Kuhn A.E., Maltzahn V., Upcin B., Karl F., Hoffmann H., Gatzner S. (2018). LOX-catalyzed collagen stabilization is a proximal cause for intrinsic resistance to chemotherapy. Oncogene.

[245] Mohammadi H., Sahai E. (2018). Mechanisms and impact of altered tumour mechanics. Nat. Cell Biol..

[246] Erler J.T., Bennewith K.L., Nicolau M., Dornhofer N., Kong C., Le Q.T., Chi J.T., Jeffrey S.S., Giaccia A.J. (2006). Lysyl oxidase is essential for hypoxia-induced metastasis. Nature.

[247] Leight J.L., Drain A.P., Weaver V.M. (2017). Extracellular Matrix Remodeling and Stiffening Modulate Tumor Phenotype and Treatment Response. Annu. Rev. Cancer Biol..

[248] Mezzenga R., Mitsi M. (2019). The Molecular Dance of Fibronectin: Conformational Flexibility Leads to Functional Versatility. Biomacromolecules.

[249] Pankov R., Yamada K.M. (2002). Fibronectin at a glance. J. Cell Sci..

[250] Baluk P., Hashizume H., McDonald D.M. (2005). Cellular abnormalities of blood vessels as targets in cancer. Curr. Opin. Genet. Dev..

[251] Dudley A.C. (2012). Tumor endothelial cells. Cold Spring Harb. Perspect. Med..

[252] Laitala A., Erler J.T. (2018). Hypoxic Signalling in Tumour Stroma. Front. Oncol..

[253] Michiels C., Tellier C., Feron O. (2016). Cycling hypoxia: A key feature of the tumor microenvironment. Biochim. Biophys. Acta.

[254] Alba-Castellon L., Olivera-Salguero R., Mestre-Farrera A., Pena R., Herrera M., Bonilla F., Casal J.I., Baulida J., Pena C., Garcia de Herreros A. (2016). Snail1-Dependent Activation of Cancer-Associated Fibroblast Controls Epithelial Tumor Cell Invasion and Metastasis. Cancer Res..

[255] Richardson A.M., Havel L.S., Koyen A.E., Konen J.M., Shupe J., Wiles W.G.t., Martin W.D., Grossniklaus H.E., Sica G., Gilbert-Ross M. (2018). Vimentin Is Required for Lung Adenocarcinoma Metastasis via Heterotypic Tumor Cell-Cancer-Associated Fibroblast Interactions during Collective Invasion. Clin. Cancer. Res..

[256] Friberg S., Nystrom A. (2015). Cancer Metastases: Early Dissemination and Late Recurrences. Cancer Growth Metastasis.

[257] Wong C.W., Lee A., Shientag L., Yu J., Dong Y., Kao G., Al-Mehdi A.B., Bernhard E.J., Muschel R.J. (2001). Apoptosis: An early event in metastatic inefficiency. Cancer Res..

[258] Fidler I.J. (1970). Metastasis: Quantitative analysis of distribution and fate of tumor emboli labeled with 125 I-5-iodo-2′-deoxyuridine. J. Natl. Cancer Inst..

[259] Massague J., Obenauf A.C. (2016). Metastatic colonization by circulating tumour cells. Nature.

[260] Guo W., Giancotti F.G. (2004). Integrin signalling during tumour progression. Nat. Rev. Mol. Cell Biol..

[261] Chen D.S., Mellman I. (2017). Elements of cancer immunity and the cancer-immune set point. Nature.

[262] Reymond N., d’Agua B.B., Ridley A.J. (2013). Crossing the endothelial barrier during metastasis. Nat. Rev. Cancer.

[263] Heikenwalder M., Lorentzen A. (2019). The role of polarisation of circulating tumour cells in cancer metastasis. Cell. Mol. Life Sci..

[264] Lorentzen A., Becker P.F., Kosla J., Saini M., Weidele K., Ronchi P., Klein C., Wolf M.J., Geist F., Seubert B. (2018). Single cell polarity in liquid phase facilitates tumour metastasis. Nat. Commun..

[265] Huang L., Cheng H.C., Isom R., Chen C.S., Levine R.A., Pauli B.U. (2008). Protein kinase Cepsilon mediates polymeric fibronectin assembly on the surface of blood-borne rat breast cancer cells to promote pulmonary metastasis. J. Biol. Chem..

[266] Aceto N., Bardia A., Miyamoto D.T., Donaldson M.C., Wittner B.S., Spencer J.A., Yu M., Pely A., Engstrom A., Zhu H. (2014). Circulating tumor cell clusters are oligoclonal precursors of breast cancer metastasis. Cell.

[267] Cheung K.J., Ewald A.J. (2016). A collective route to metastasis: Seeding by tumor cell clusters. Science.

[268] Hong Y., Fang F., Zhang Q. (2016). Circulating tumor cell clusters: What we know and what we expect (Review). Int. J. Oncol..

[269] Yin T., Getsios S., Caldelari R., Kowalczyk A.P., Muller E.J., Jones J.C., Green K.J. (2005). Plakoglobin suppresses keratinocyte motility through both cell-cell adhesion-dependent and -independent mechanisms. Proc. Natl. Acad. Sci. USA.

[270] Todorovic V., Desai B.V., Patterson M.J., Amargo E.V., Dubash A.D., Yin T., Jones J.C., Green K.J. (2010). Plakoglobin regulates cell motility through Rho- and fibronectin-dependent Src signaling. J. Cell Sci..

[271] Aceto N., Toner M., Maheswaran S., Haber D.A. (2015). En Route to Metastasis: Circulating Tumor Cell Clusters and Epithelial-to-Mesenchymal Transition. Trends Cancer.

[272] Stegner D., Dutting S., Nieswandt B. (2014). Mechanistic explanation for platelet contribution to cancer metastasis. Thromb. Res..

[273] Gil-Bernabe A.M., Lucotti S., Muschel R.J. (2013). Coagulation and metastasis: What does the experimental literature tell us?. Br. J. Haematol..

[274] Schick P.K., Wojenski C.M., He X., Walker J., Marcinkiewicz C., Niewiarowski S. (1998). Integrins involved in the adhesion of megakaryocytes to fibronectin and fibrinogen. Blood.

[275] Li J., Ai Y., Wang L., Bu P., Sharkey C.C., Wu Q., Wun B., Roy S., Shen X., King M.R. (2016). Targeted drug delivery to circulating tumor cells via platelet membrane-functionalized particles. Biomaterials.

[276] Griggs L.A., Hassan N.T., Malik R.S., Griffin B.P., Martinez B.A., Elmore L.W., Lemmon C.A. (2017). Fibronectin fibrils regulate TGF-beta1-induced Epithelial-Mesenchymal Transition. Matrix Biol..

[277] Massague J. (2008). TGFbeta in Cancer. Cell.

[278] Tesfamariam B. (2016). Involvement of platelets in tumor cell metastasis. Pharmacol. Ther..

[279] Matrone M.A., Whipple R.A., Balzer E.M., Martin S.S. (2010). Microtentacles tip the balance of cytoskeletal forces in circulating tumor cells. Cancer Res..

[280] Whipple R.A., Balzer E.M., Cho E.H., Matrone M.A., Yoon J.R., Martin S.S. (2008). Vimentin filaments support extension of tubulin-based microtentacles in detached breast tumor cells. Cancer Res..

[281] Cheng F., Shen Y., Mohanasundaram P., Lindstrom M., Ivaska J., Ny T., Eriksson J.E. (2016). Vimentin coordinates fibroblast proliferation and keratinocyte differentiation in wound healing via TGF-beta-Slug signaling. Proc. Natl. Acad. Sci. USA.

[282] Eckes B., Dogic D., Colucci-Guyon E., Wang N., Maniotis A., Ingber D., Merckling A., Langa F., Aumailley M., Delouvee A. (1998). Impaired mechanical stability, migration and contractile capacity in vimentin-deficient fibroblasts. J. Cell Sci..

[283] Kim J., Jang J., Yang C., Kim E.J., Jung H., Kim C. (2016). Vimentin filament controls integrin alpha5beta1-mediated cell adhesion by binding to integrin through its Ser38 residue. FEBS Lett..

[284] Ivaska J., Vuoriluoto K., Huovinen T., Izawa I., Inagaki M., Parker P.J. (2005). PKCepsilon-mediated phosphorylation of vimentin controls integrin recycling and motility. Embo J..

[285] Dauphin M., Barbe C., Lemaire S., Nawrocki-Raby B., Lagonotte E., Delepine G., Birembaut P., Gilles C., Polette M. (2013). Vimentin expression predicts the occurrence of metastases in non small cell lung carcinomas. Lung Cancer.

[286] Morvan M.G., Lanier L.L. (2016). NK cells and cancer: You can teach innate cells new tricks. Nat. Rev. Cancer.

[287] Nieswandt B., Hafner M., Echtenacher B., Mannel D.N. (1999). Lysis of tumor cells by natural killer cells in mice is impeded by platelets. Cancer Res..

[288] Palumbo J.S., Talmage K.E., Massari J.V., La Jeunesse C.M., Flick M.J., Kombrinck K.W., Jirouskova M., Degen J.L. (2005). Platelets and fibrin(ogen) increase metastatic potential by impeding natural killer cell-mediated elimination of tumor cells. Blood.

[289] Placke T., Salih H.R., Kopp H.G. (2012). GITR ligand provided by thrombopoietic cells inhibits NK cell antitumor activity. J. Immunol..

[290] Ferjancic S., Gil-Bernabe A.M., Hill S.A., Allen P.D., Richardson P., Sparey T., Savory E., McGuffog J., Muschel R.J. (2013). VCAM-1 and VAP-1 recruit myeloid cells that promote pulmonary metastasis in mice. Blood.

[291] Van der Weyden L., Arends M.J., Campbell A.D., Bald T., Wardle-Jones H., Griggs N., Velasco-Herrera M.D., Tuting T., Sansom O.J., Karp N.A. (2017). Genome-wide in vivo screen identifies novel host regulators of metastatic colonization. Nature.

[292] Szczerba B.M., Castro-Giner F., Vetter M., Krol I., Gkountela S., Landin J., Scheidmann M.C., Donato C., Scherrer R., Singer J. (2019). Neutrophils escort circulating tumour cells to enable cell cycle progression. Nature.

[293] Coffelt S.B., Wellenstein M.D., de Visser K.E. (2016). Neutrophils in cancer: Neutral no more. Nat. Rev. Cancer.

[294] Coffelt S.B., Kersten K., Doornebal C.W., Weiden J., Vrijland K., Hau C.S., Verstegen N.J.M., Ciampricotti M., Hawinkels L., Jonkers J. (2015). IL-17-producing gammadelta T cells and neutrophils conspire to promote breast cancer metastasis. Nature.

[295] Spiegel A., Brooks M.W., Houshyar S., Reinhardt F., Ardolino M., Fessler E., Chen M.B., Krall J.A., DeCock J., Zervantonakis I.K. (2016). Neutrophils Suppress Intraluminal NK Cell-Mediated Tumor Cell Clearance and Enhance Extravasation of Disseminated Carcinoma Cells. Cancer Discov..

[296] Mazel M., Jacot W., Pantel K., Bartkowiak K., Topart D., Cayrefourcq L., Rossille D., Maudelonde T., Fest T., Alix-Panabieres C. (2015). Frequent expression of PD-L1 on circulating breast cancer cells. Mol. Oncol..

[297] Nicolazzo C., Raimondi C., Mancini M., Caponnetto S., Gradilone A., Gandini O., Mastromartino M., Del Bene G., Prete A., Longo F. (2016). Monitoring PD-L1 positive circulating tumor cells in non-small cell lung cancer patients treated with the PD-1 inhibitor Nivolumab. Sci. Rep..

[298] Alsuliman A., Colak D., Al-Harazi O., Fitwi H., Tulbah A., Al-Tweigeri T., Al-Alwan M., Ghebeh H. (2015). Bidirectional crosstalk between PD-L1 expression and epithelial to mesenchymal transition: Significance in claudin-low breast cancer cells. Mol. Cancer.

[299] Hou P., Li L., Chen F., Chen Y., Liu H., Li J., Bai J., Zheng J. (2018). PTBP3-Mediated Regulation of ZEB1 mRNA Stability Promotes Epithelial-Mesenchymal Transition in Breast Cancer. Cancer Res..

[300] Perdigao-Henriques R., Petrocca F., Altschuler G., Thomas M.P., Le M.T., Tan S.M., Hide W., Lieberman J. (2016). miR-200 promotes the mesenchymal to epithelial transition by suppressing multiple members of the Zeb2 and Snail1 transcriptional repressor complexes. Oncogene.

[301] Kun-Peng Z., Chun-Lin Z., Xiao-Long M., Lei Z. (2019). Fibronectin-1 modulated by the long noncoding RNA OIP5-AS1/miR-200b-3p axis contributes to doxorubicin resistance of osteosarcoma cells. J. Cell. Physiol..

[302] Lin T.-C., Liao Y.-C., Chang W.-T., Yang C.-H., Cheng L.-H., Cheng M., Cheng H.-C. (2018). The Establishment of a Lung Colonization Assay for Circulating Tumor Cell Visualization in Lung Tissues. JoVE.

[303] Huang R.L., Teo Z., Chong H.C., Zhu P., Tan M.J., Tan C.K., Lam C.R., Sng M.K., Leong D.T., Tan S.M. (2011). ANGPTL4 modulates vascular junction integrity by integrin signaling and disruption of intercellular VE-cadherin and claudin-5 clusters. Blood.

[304] Padua D., Zhang X.H., Wang Q., Nadal C., Gerald W.L., Gomis R.R., Massague J. (2008). TGFbeta primes breast tumors for lung metastasis seeding through angiopoietin-like 4. Cell.

[305] Shen C.J., Chan S.H., Lee C.T., Huang W.C., Tsai J.P., Chen B.K. (2017). Oleic acid-induced ANGPTL4 enhances head and neck squamous cell carcinoma anoikis resistance and metastasis via up-regulation of fibronectin. Cancer Lett..

[306] Tichet M., Prod’Homme V., Fenouille N., Ambrosetti D., Mallavialle A., Cerezo M., Ohanna M., Audebert S., Rocchi S., Giacchero D. (2015). Tumour-derived SPARC drives vascular permeability and extravasation through endothelial VCAM1 signalling to promote metastasis. Nat. Commun..

[307] Strilic B., Yang L., Albarran-Juarez J., Wachsmuth L., Han K., Muller U.C., Pasparakis M., Offermanns S. (2016). Tumour-cell-induced endothelial cell necroptosis via death receptor 6 promotes metastasis. Nature.

[308] Kaczmarek A., Vandenabeele P., Krysko D.V. (2013). Necroptosis: The release of damage-associated molecular patterns and its physiological relevance. Immunity.

[309] Langer H.F., Orlova V.V., Xie C., Kaul S., Schneider D., Lonsdorf A.S., Fahrleitner M., Choi E.Y., Dutoit V., Pellegrini M. (2011). A novel function of junctional adhesion molecule-C in mediating melanoma cell metastasis. Cancer Res..

[310] Jouve N., Bachelier R., Despoix N., Blin M.G., Matinzadeh M.K., Poitevin S., Aurrand-Lions M., Fallague K., Bardin N., Blot-Chabaud M. (2015). CD146 mediates VEGF-induced melanoma cell extravasation through FAK activation. Int. J. Cancer.

[311] Melnikova V.O., Balasubramanian K., Villares G.J., Dobroff A.S., Zigler M., Wang H., Petersson F., Price J.E., Schroit A., Prieto V.G. (2009). Crosstalk between protease-activated receptor 1 and platelet-activating factor receptor regulates melanoma cell adhesion molecule (MCAM/MUC18) expression and melanoma metastasis. J. Biol. Chem..

[312] Gassmann P., Haier J., Schluter K., Domikowsky B., Wendel C., Wiesner U., Kubitza R., Engers R., Schneider S.W., Homey B. (2009). CXCR4 regulates the early extravasation of metastatic tumor cells in vivo. Neoplasia.

[313] Gomis R.R., Gawrzak S. (2017). Tumor cell dormancy. Mol. Oncol..

[314] Aguirre-Ghiso J.A. (2018). How dormant cancer persists and reawakens. Science.

[315] Holmgren L., O’Reilly M.S., Folkman J. (1995). Dormancy of micrometastases: Balanced proliferation and apoptosis in the presence of angiogenesis suppression. Nat. Med..

[316] Krall J.A., Reinhardt F., Mercury O.A., Pattabiraman D.R., Brooks M.W., Dougan M., Lambert A.W., Bierie B., Ploegh H.L., Dougan S.K. (2018). The systemic response to surgery triggers the outgrowth of distant immune-controlled tumors in mouse models of dormancy. Sci. Transl. Med..

[317] Ghajar C.M. (2015). Metastasis prevention by targeting the dormant niche. Nat. Rev. Cancer.

[318] Mittal V. (2018). Epithelial Mesenchymal Transition in Tumor Metastasis. Annu. Rev. Pathol..

[319] Stoletov K., Kato H., Zardouzian E., Kelber J., Yang J., Shattil S., Klemke R. (2010). Visualizing extravasation dynamics of metastatic tumor cells. J. Cell Sci..

[320] Shibue T., Brooks M.W., Inan M.F., Reinhardt F., Weinberg R.A. (2012). The outgrowth of micrometastases is enabled by the formation of filopodium-like protrusions. Cancer Discov..

[321] Harper K.L., Sosa M.S., Entenberg D., Hosseini H., Cheung J.F., Nobre R., Avivar-Valderas A., Nagi C., Girnius N., Davis R.J. (2016). Mechanism of early dissemination and metastasis in Her2(^+^) mammary cancer. Nature.

[322] Tarin D., Thompson E.W., Newgreen D.F. (2005). The fallacy of epithelial mesenchymal transition in neoplasia. Cancer Res..

[323] Thompson E.W., Newgreen D.F., Tarin D. (2005). Carcinoma invasion and metastasis: A role for epithelial-mesenchymal transition?. Cancer Res..

[324] Yates C. (2011). Prostate tumor cell plasticity: A consequence of the microenvironment. Adv. Exp. Med. Biol..

[325] Prudkin L., Liu D.D., Ozburn N.C., Sun M., Behrens C., Tang X., Brown K.C., Bekele B.N., Moran C., Wistuba I.I. (2009). Epithelial-to-mesenchymal transition in the development and progression of adenocarcinoma and squamous cell carcinoma of the lung. Mod. Pathol..

[326] Chaffer C.L., Thompson E.W., Williams E.D. (2007). Mesenchymal to epithelial transition in development and disease. Cells Tissues Organs.

[327] Yao D., Dai C., Peng S. (2011). Mechanism of the mesenchymal–epithelial transition and its relationship with metastatic tumor formation. Mol. Cancer Res..

[328] Brabletz T. (2012). EMT and MET in metastasis: Where are the cancer stem cells?. Cancer Cell.

[329] Nieto M.A. (2013). Epithelial plasticity: A common theme in embryonic and cancer cells. Science.

[330] Ocana O.H., Corcoles R., Fabra A., Moreno-Bueno G., Acloque H., Vega S., Barrallo-Gimeno A., Cano A., Nieto M.A. (2012). Metastatic colonization requires the repression of the epithelial-mesenchymal transition inducer Prrx1. Cancer Cell.

[331] Li J., Yang Z., Chen Z., Bao Y., Zhang H., Fang X., Yang W. (2016). ATF3 suppresses ESCC via downregulation of ID1. Oncol. Lett..

[332] Stanisavljevic J., Porta-de-la-Riva M., Batlle R., de Herreros A.G., Baulida J. (2011). The p65 subunit of NF-kappaB and PARP1 assist Snail1 in activating fibronectin transcription. J. Cell Sci..

[333] Tran H.D., Luitel K., Kim M., Zhang K., Longmore G.D., Tran D.D. (2014). Transient SNAIL1 expression is necessary for metastatic competence in breast cancer. Cancer Res..

[334] Stankic M., Pavlovic S., Chin Y., Brogi E., Padua D., Norton L., Massague J., Benezra R. (2013). TGF-beta-Id1 signaling opposes Twist1 and promotes metastatic colonization via a mesenchymal-to-epithelial transition. Cell Rep..

[335] Sharma S., Xing F., Liu Y., Wu K., Said N., Pochampally R., Shiozawa Y., Lin H.K., Balaji K.C., Watabe K. (2016). Secreted Protein Acidic and Rich in Cysteine (SPARC) Mediates Metastatic Dormancy of Prostate Cancer in Bone. J. Biol. Chem..

[336] Lelekakis M., Moseley J.M., Martin T.J., Hards D., Williams E., Ho P., Lowen D., Javni J., Miller F.R., Slavin J. (1999). A novel orthotopic model of breast cancer metastasis to bone. Clin. Exp. Metastasis.

[337] Khanna C., Hunter K. (2005). Modeling metastasis in vivo. Carcinogenesis.

[338] Lou Y., Preobrazhenska O., auf dem Keller U., Sutcliffe M., Barclay L., McDonald P.C., Roskelley C., Overall C.M., Dedhar S. (2008). Epithelial-mesenchymal transition (EMT) is not sufficient for spontaneous murine breast cancer metastasis. Dev. Dyn..

[339] Yang J., Mani S.A., Donaher J.L., Ramaswamy S., Itzykson R.A., Come C., Savagner P., Gitelman I., Richardson A., Weinberg R.A. (2004). Twist, a master regulator of morphogenesis, plays an essential role in tumor metastasis. Cell.

[340] Eckhardt B.L., Parker B.S., van Laar R.K., Restall C.M., Natoli A.L., Tavaria M.D., Stanley K.L., Sloan E.K., Moseley J.M., Anderson R.L. (2005). Genomic analysis of a spontaneous model of breast cancer metastasis to bone reveals a role for the extracellular matrix. Mol. Cancer Res..

[341] Shamir E.R., Pappalardo E., Jorgens D.M., Coutinho K., Tsai W.T., Aziz K., Auer M., Tran P.T., Bader J.S., Ewald A.J. (2014). Twist1-induced dissemination preserves epithelial identity and requires E-cadherin. J. Cell Biol..

[342] Padmanaban V., Krol I., Suhail Y., Szczerba B.M., Aceto N., Bader J.S., Ewald A.J. (2019). E-cadherin is required for metastasis in multiple models of breast cancer. Nature.

[343] Esposito M., Mondal N., Greco T.M., Wei Y., Spadazzi C., Lin S.C., Zheng H., Cheung C., Magnani J.L., Lin S.H. (2019). Bone vascular niche E-selectin induces mesenchymal–epithelial transition and Wnt activation in cancer cells to promote bone metastasis. Nat. Cell Biol..

[344] Del Pozo Martin Y., Park D., Ramachandran A., Ombrato L., Calvo F., Chakravarty P., Spencer-Dene B., Derzsi S., Hill C.S., Sahai E. (2015). Mesenchymal Cancer Cell-Stroma Crosstalk Promotes Niche Activation, Epithelial Reversion, and Metastatic Colonization. Cell Rep..

[345] Asiedu M.K., Beauchamp-Perez F.D., Ingle J.N., Behrens M.D., Radisky D.C., Knutson K.L. (2014). AXL induces epithelial-to-mesenchymal transition and regulates the function of breast cancer stem cells. Oncogene.

[346] Kitamura T., Qian B.Z., Pollard J.W. (2015). Immune cell promotion of metastasis. Nat. Rev. Immunol..

[347] Hoshino A., Costa-Silva B., Shen T.L., Rodrigues G., Hashimoto A., Tesic Mark M., Molina H., Kohsaka S., Di Giannatale A., Ceder S. (2015). Tumour exosome integrins determine organotropic metastasis. Nature.

[348] Price J.E., Polyzos A., Zhang R.D., Daniels L.M. (1990). Tumorigenicity and metastasis of human breast carcinoma cell lines in nude mice. Cancer Res..

[349] Sharma R., Sharma R., Khaket T.P., Dutta C., Chakraborty B., Mukherjee T.K. (2017). Breast cancer metastasis: Putative therapeutic role of vascular cell adhesion molecule-1. Cell. Oncol..

[350] Sleeman J.P., Nazarenko I., Thiele W. (2011). Do all roads lead to Rome? Routes to metastasis development. Int. J. Cancer.

[351] Villa A., Trachsel E., Kaspar M., Schliemann C., Sommavilla R., Rybak J.N., Rosli C., Borsi L., Neri D. (2008). A high-affinity human monoclonal antibody specific to the alternatively spliced EDA domain of fibronectin efficiently targets tumor neo-vasculature in vivo. Int. J. Cancer.

[352] Tijink B.M., Neri D., Leemans C.R., Budde M., Dinkelborg L.M., Stigter-van Walsum M., Zardi L., van Dongen G.A. (2006). Radioimmunotherapy of head and neck cancer xenografts using 131I-labeled antibody L19-SIP for selective targeting of tumor vasculature. J. Nucl. Med..

[353] Ogura A., Konishi T., Cunningham C., Garcia-Aguilar J., Iversen H., Toda S., Lee I.K., Lee H.X., Uehara K., Lee P. (2019). Neoadjuvant (Chemo)radiotherapy With Total Mesorectal Excision Only Is Not Sufficient to Prevent Lateral Local Recurrence in Enlarged Nodes: Results of the Multicenter Lateral Node Study of Patients With Low cT3/4 Rectal Cancer. J. Clin. Oncol..

[354] Leong T. (2018). A CRITICal period for chemoradiotherapy in gastric cancer. Lancet Oncol..

[355] Postow M.A., Callahan M.K., Wolchok J.D. (2015). Immune Checkpoint Blockade in Cancer Therapy. J. Clin. Oncol..

[356] Yoshida A., Lee E.K., Diehl J.A. (2016). Induction of Therapeutic Senescence in Vemurafenib-Resistant Melanoma by Extended Inhibition of CDK4/6. Cancer Res..

[357] Di Mitri D., Mirenda M., Vasilevska J., Calcinotto A., Delaleu N., Revandkar A., Gil V., Boysen G., Losa M., Mosole S. (2019). Re-education of Tumor-Associated Macrophages by CXCR2 Blockade Drives Senescence and Tumor Inhibition in Advanced Prostate Cancer. Cell Rep..

[358] Cho H., Seo Y., Loke K.M., Kim S.W., Oh S.M., Kim J.H., Soh J., Kim H.S., Lee H., Kim J. (2018). Cancer-Stimulated CAFs Enhance Monocyte Differentiation and Protumoral TAM Activation via IL6 and GM-CSF Secretion. Clin. Cancer. Res..

[359] Prakash J. (2016). Cancer-Associated Fibroblasts: Perspectives in Cancer Therapy. Trends Cancer.

[360] De Vlieghere E., Verset L., Demetter P., Bracke M., De Wever O. (2015). Cancer-associated fibroblasts as target and tool in cancer therapeutics and diagnostics. Virchows Arch..

[361] Komohara Y., Fujiwara Y., Ohnishi K., Takeya M. (2016). Tumor-associated macrophages: Potential therapeutic targets for anti-cancer therapy. Adv. Drug Deliv. Rev..

[362] Calon A., Lonardo E., Berenguer-Llergo A., Espinet E., Hernando-Momblona X., Iglesias M., Sevillano M., Palomo-Ponce S., Tauriello D.V., Byrom D. (2015). Stromal gene expression defines poor-prognosis subtypes in colorectal cancer. Nat. Genet..

[363] Komohara Y., Morita T., Annan D.A., Horlad H., Ohnishi K., Yamada S., Nakayama T., Kitada S., Suzu S., Kinoshita I. (2015). The Coordinated Actions of TIM-3 on Cancer and Myeloid Cells in the Regulation of Tumorigenicity and Clinical Prognosis in Clear Cell Renal Cell Carcinomas. Cancer Immunol. Res..

